# A Combined Comparative Transcriptomic, Metabolomic, and Anatomical Analyses of Two Key Domestication Traits: Pod Dehiscence and Seed Dormancy in Pea (*Pisum* sp.)

**DOI:** 10.3389/fpls.2017.00542

**Published:** 2017-04-25

**Authors:** Iveta Hradilová, Oldřich Trněný, Markéta Válková, Monika Cechová, Anna Janská, Lenka Prokešová, Khan Aamir, Nicolas Krezdorn, Björn Rotter, Peter Winter, Rajeev K. Varshney, Aleš Soukup, Petr Bednář, Pavel Hanáček, Petr Smýkal

**Affiliations:** ^1^Department of Botany, Palacký University in OlomoucOlomouc, Czechia; ^2^Department of Plant Biology, Mendel University in BrnoBrno, Czechia; ^3^Agricultural Research, Ltd.Troubsko, Czechia; ^4^Department of Analytical Chemistry, Regional Centre of Advanced Technologies and Materials, Palacký University in OlomoucOlomouc, Czechia; ^5^Faculty of Science, Palacký University in OlomoucOlomouc, Czechia; ^6^Department of Experimental Plant Biology, Charles UniversityPrague, Czechia; ^7^Department of Crop Science, Breeding and Plant Medicine, Mendel University in BrnoBrno, Czechia; ^8^Research Program-Genetic Gains, ICRISATHyderabad, India; ^9^GenXProFrankfurt, Germany

**Keywords:** domestication, legumes, pea (*Pisum sativum*), metabolites, pod dehiscence, seed dormancy, seed coat, transcriptomics

## Abstract

The origin of the agriculture was one of the turning points in human history, and a central part of this was the evolution of new plant forms, domesticated crops. Seed dispersal and germination are two key traits which have been selected to facilitate cultivation and harvesting of crops. The objective of this study was to analyze anatomical structure of seed coat and pod, identify metabolic compounds associated with water-impermeable seed coat and differentially expressed genes involved in pea seed dormancy and pod dehiscence. Comparative anatomical, metabolomics, and transcriptomic analyses were carried out on wild dormant, dehiscent *Pisum elatius* (JI64, VIR320) and cultivated, indehiscent *Pisum sativum* non-dormant (JI92, Cameor) and recombinant inbred lines (RILs). Considerable differences were found in texture of testa surface, length of macrosclereids, and seed coat thickness. Histochemical and biochemical analyses indicated genotype related variation in composition and heterogeneity of seed coat cell walls within macrosclereids. Liquid chromatography–electrospray ionization/mass spectrometry and Laser desorption/ionization–mass spectrometry of separated seed coats revealed significantly higher contents of proanthocyanidins (dimer and trimer of gallocatechin), quercetin, and myricetin rhamnosides and hydroxylated fatty acids in dormant compared to non-dormant genotypes. Bulk Segregant Analysis coupled to high throughput RNA sequencing resulted in identification of 770 and 148 differentially expressed genes between dormant and non-dormant seeds or dehiscent and indehiscent pods, respectively. The expression of 14 selected dormancy-related genes was studied by qRT-PCR. Of these, expression pattern of four genes: porin (MACE-S082), peroxisomal membrane PEX14-like protein (MACE-S108), 4-coumarate CoA ligase (MACE-S131), and UDP-glucosyl transferase (MACE-S139) was in agreement in all four genotypes with Massive analysis of cDNA Ends (MACE) data. In case of pod dehiscence, the analysis of two candidate genes (*SHATTERING* and *SHATTERPROOF*) and three out of 20 MACE identified genes (MACE-P004, MACE-P013, MACE-P015) showed down-expression in dorsal and ventral pod suture of indehiscent genotypes. Moreover, MACE-P015, the homolog of peptidoglycan-binding domain or proline-rich extensin-like protein mapped correctly to predicted *Dpo1* locus on PsLGIII. This integrated analysis of the seed coat in wild and cultivated pea provides new insight as well as raises new questions associated with domestication and seed dormancy and pod dehiscence.

## Introduction

The origin of the agriculture was one of key points in human history, and a central part of this was the evolution of new plant forms, domesticated crops (Meyer et al., 2012; Fuller et al., 2014). The transformation of wild plants into crop plants can be viewed as an accelerated evolution, representing adaptations to cultivation and human harvesting, accompanied by genetic changes (Lenser and Theißen, 2013; Olsen and Wendel, 2013; Shi and Lai, 2015). Common set of traits have been recorded for unrelated crops (Hammer, 1984; Zohary and Hopf, 2000; Lenser and Theißen, 2013). These include loss of germination inhibition and loss of natural seed dispersal (Fuller and Allaby, 2009). The identity of some responsible genes has been revealed (reviewed in Meyer and Purugganan, 2013) through association mapping and genome sequencing, for example in soybean (Zhou et al., 2015), chickpea (Bajaj et al., 2015; Kujur et al., 2015), and common bean (Schmutz et al., 2014).

Members of the *Fabaceae* family have been domesticated in parallel with cereals (Smartt, 1990; Zohary and Hopf, 2000) or possibly even earlier (Kislev and Bar-Yosef, 1988) resulting in largest number of domesticates per plant family (Smýkal et al., 2015). Despite of crucial position of legumes, as protein crops, in human diet as well as crop rotation systems (Foyer et al., 2016), comparably little is known on their domestication. Pea (*Pisum sativum* L.) is one of the world's oldest domesticated crops and is still globally important grain legume crop (Smýkal et al., 2012, 2015). Experimental cultivation of wild peas have demonstrated that both seed dormancy and pod dehiscence cause poor crop establishment via reduced germination as well as dramatic yield losses via seed shattering (Abbo et al., 2011). The loss of fruit shattering has been under selection in the most seed crops, to facilitate seed harvest (Fuller and Allaby, 2009; Purugganan and Fuller, 2009), while in wild plants, shattering is a fundamental trait to assure seed dispersal (Bennett et al., 2011). Orthologous genes and functions were found to be conserved for seed shattering mechanisms between mono and dicotyledonous plants (Konishi et al., 2006). Recently, two genes have been identified to be involved in pod dehiscence in soybean. One of them is the dirigent-like protein (*Pdh1*) promoting pod dehiscence by increasing the torsion of dried pod walls, which serves as a driving force for pod dehiscence under low humidity (Funatsuki et al., 2014). The functional gene *Pdh1* was highly expressed in the lignin-rich inner sclerenchyma of pod walls. Yet, another NAC family gene SHATTERING1-5 (Dong et al., 2014) activates secondary wall biosynthesis and promotes the significant thickening of fiber cap cells of the pod ventral suture secondary walls. The differences between wild and cultivated soybean is within promoter region and subsequently expression level (Dong et al., 2014).

Timing of seed germination is one of the key steps in plant life. Seed dormancy is considered as a block to the completion of germination of an intact viable seed under favorable conditions (Baskin and Baskin, 2004; Weitbrecht et al., 2011). In the wild, many seeds will only germinate after certain conditions have passed, or after the seed coat is physically disrupted (Bewley, 1997; Baskin et al., 2000; Finch-Savage and Leubner-Metzger, 2006; Bewley et al., 2013). In contrast, crops were selected to germinate as soon as they are wet and planted (Weitbrecht et al., 2011). Moreover, easy seed imbibition has crucial role in cooking ability of most grain legumes. Hence, reducing seed coat thickness led to a concurrent reduction of seed coat impermeability during the domestication (Smýkal et al., 2014). Seed dormancy had played a significant role in evolution and adaptation of plants, as it determines the outset of a new generation (Nonogaki, 2014; Smýkal et al., 2014). A diverse dormancy mechanisms has evolved in keeping with the diversity of climates and habitats (Nikolaeva, 1969; Baskin and Baskin, 2004; Finch-Savage and Leubner-Metzger, 2006). In contrast to hormone mediated seed dormancy extensively studied in *Arabidopsis* or cereals, we have very little knowledge on physical dormancy, as found in legumes (Baskin and Baskin, 2004; Graeber et al., 2012; Radchuk and Borisjuk, 2014). Although hard-seededness was largely overcome in all domesticated grain legumes except of fodder legumes (Werker et al., 1979; Smartt, 1990; Weeden, 2007), it appears in lentil or soybean depending on the cultivation conditions. Physical seed dormancy is caused by one or more water-impermeable cell layers in seed coat (Baskin et al., 2000; Koizumi et al., 2008; Weitbrecht et al., 2011; Radchuk and Borisjuk, 2014; Smýkal et al., 2014). Numerous *transparent testa* (*tt*) and *tannin deficient seed* (*tds*) mutants (Appelhagen et al., 2014) indicates the important role of proanthocyanidins and flavonoid pigments in *Arabidopsis* (Graeber et al., 2012) and *Medicago* (Liu et al., 2014) testa development. In *Arabidopsis* and *Melilotus*, seed permeability is altered due to in mutation in extracellular lipid biosynthesis (Beisson et al., 2007). Similarly, in the *M. truncatula* transcriptomic data set (Verdier et al., 2013a), four of 12 Glycerol-3-phosphate acyltransferases (GPAT) genes were identified as putative orthologs of those reported in soybean (Ranathunge et al., 2010). Furthermore, cells of the outer integument in *M. truncatula* and pea showed abundant accumulation of polyphenolic compounds; which upon oxidation may impact seed permeability (Marbach and Mayer, 1974; Werker et al., 1979; Moïse et al., 2005). Seed dormancy was identified as monogenic trait in mungbean (Isemura et al., 2012); while six QTLs were detected in yardlong and rice bean (Kongjaimun et al., 2012). In pea, Weeden (2007) has identified two to three loci involved in seed dormancy, via testa thickness and structure of testa surface. Two genes involved in seed coat water permeability were recently identified in soybean. One of them, *GmHs1-1*, encodes a calcineurin-like metallophosphoesterase transmembrane protein, which is primarily expressed in the Malpighian layer (macrosclereids) of the seed coat and is associated with calcium content (Sun et al., 2015). Independently of this, *qHS1*, a quantitative trait locus for hardseededness in soybean, was identified as endo-1,4-β-glucanase (Jang et al., 2015). This genes seems to be involved in the accumulation of β-1,4-glucan derivatives that reinforce the impermeability of seed coats in soybean. Interestingly, both genes are positioned closely to each other of soybean chromosome 2.

Development of pea and particularly model legume *Medicago truncatula* seeds have been well-characterized at anatomical (Hedley et al., 1986; Wang and Grusak, 2005) and also transcriptomic and proteomic levels (Gallardo et al., 2007; Verdier et al., 2013a). RNA sequencing (RNA-seq) was used to study changes in gene expression, including *M. truncatula* (Benedito et al., 2008), *Medicago sativa* (Zhang et al., 2015), soybean (Severin et al., 2010; Patil et al., 2015), faba bean (Kaur et al., 2012), *Lotus japonicus* (Verdier et al., 2013b) and chickpea (Pradhan et al., 2014). In pea, transcriptome studies involved vegetative tissues (Franssen et al., 2011), including pods and seeds (Kaur et al., 2012; Duarte et al., 2014; Liu et al., 2015; Sudheesh et al., 2015), and nodules (Zhukov et al., 2015). Seed coat transcriptome of pea cultivars was analyzed in relation to proanthocyanidin pathway (Ferraro et al., 2014) and seed aging (Chen et al., 2013). Moreover, there is pea RNA-seq gene atlas for 20 cDNA libraries including different developmental stages and nutritive conditions (Alves-Carvalho et al., 2015). Comparative transcriptomics study in relation to domestication trait was conducted recently by Zou et al. (2015) in relation to glume and threshing in wheat. Some of the down-regulated genes in domesticated wheat were related to the biosynthetic pathways that apparently define the mechanical strength of the glumes, such as cell wall, lignin, pectin, and wax biosynthesis. Several of so far identified genes underlying key domestication traits (reviewed in Meyer and Purugganan, 2013) are regulated at transcriptional level with altered spatial and temporal expression, such as seed-shattering (*qSH1*) locus disrupting the development of the abscission zone between grains and pedicles in rice (Konishi et al., 2006) or *teosinte branched* (*tb1*) gene causing single stem growth in maize crop (Doebley et al., 1997).

In the present study, we used comparative transcriptomic, anatomical, and metabolite analysis to detect the differences in gene expression, seed coat structure, and metabolites composition between wild and domesticated pea seed coats in relation to one of the two key domestication traits: seed coat and transcriptomic and anatomical analyses of pod dehiscence.

## Materials and methods

### Plant material

Four parental genotypes included wild *P. elatius* JI64 from Turkey and cultivated Afghan landrace *P. sativum* JI92 both from John Innes Pisum Collection (Norwich, UK); wild *P. elatius* VIR320 (Bogdanova et al., 2012) from Vavilov Institute Research of Plant Industry (St. Petersburg, Russia) and cultivated *P. sativum* cv. Cameor from INRA France. Furthermore, 126 F_5:6_ recombinant inbred lines (RILs) derived from JI64 and JI92 cross (North et al., 1989) were used to establish respective phenotypically contrasting (dormant vs. non-dormant, dehiscent vs. indehiscent) bulks. *P. elatius* VIR320 differs from other wild peas in relation to the absence of gritty and testa pigmentation, possibly as the result of being either semi-domesticate or hybrid between wild and cultivated pea with unknown origin (Bogdanova et al., 2012).

### Seed water uptake and germination assays

The seeds of four parental and 126 RILs of F_6_ generation of JI64 (wild) × JI92 (cultivated) and reciprocal (RILs) were harvested from glasshouse grown plants (February–May 2015). Twenty five seeds per line were incubated in petri dishes (9 cm diameter) over two layers of medium speed qualitative filter papers (Whatman, grade 1) wetted with 3 ml of tap water and incubated in a 25°C incubator with darkness. Imbibition was scored at 24 h intervals based on changes in seed swelling and germination was determined based on the radicle breaking through seed coat. The percentage, Mean germination time (MGT), Timson index (TI), and Coefficient of Velocity (CV) were calculated over 7 days period. We have used these various mathematical measurements in order to more precisely describe germination process as shown by Ranal and Santana (2006).

### Determination of pod dehiscence

Pod dehiscence was measured either by direct observation of the pods on the plant or by drying harvested pods at room temperature (Weeden et al., 2002; Weeden, 2007). In case of parental lines JI92 (domesticated, indehiscent pod) and JI64 (wild, dehiscent pod) are dehiscence/indehiscence obvious after pod maturing. On the other hand evaluation of RILs was difficult in some cases, as slight pressure on pods by fingers is necessary for opening. If slight pressure was enough to complete fruit opening the line was evaluated as dehiscent, if not this line was evaluated as indehiscent.

### Anatomical analyses

Samples of seed coat (JI64, VIR320, Cameor and JI92; at least five seeds per genotype) were dissected from dry seed and saturated with 2% sucrose solution under vacuum. Equal volume of cryo-gel (Cryomatrix Shandon) was added to samples and shake overnight. Saturated samples were mounted into cryo-gel on the alum chuck, frozen down to −25°C and cut in cryotome (Shandon SME, Astmoor, UK) into 12 μm transversal section (Soukup and Tylová, 2014). Sections were stained with toluidine blue (0.01%, w/v in water), alcian blue (0.1%, w/v in 3% acetic acid), aniline blue fluorochrome (Sirofluor; 0.01%, w/v in 100 mM K_2_HPO_4_ with pH 9), or Sudan Red 7B (0.01%, w/v) according to Soukup (2014). The presence of proanthocyanidins was evaluated by staining with vanillin (Gardner, 1975) and DMACA (Li et al., 1996). Callose immunodetection was performed according to Soukup (2014) using primary antibody toward (1,3)-β-glucan (1:100; Biosupplies Australia PTY Ltd) and anti-mouse IgG Alexa Fluor 488 secondary antibody (1:500; Invitrogen). Control samples were processed without the primary antibody. Sections were observed with an Olympus BX 51 microscope (Olympus Corp., Tokyo, Japan) in bright field, blue (Olympus WB filter—callose immunodetection) or UV (Olympus WU filter—aniline blue fluorochrome and DMACA) excitation. Unstained control sections were surveyed in bright field or UV-excited autofluorescence. Figures were documented with an Apogee U4000 digital camera (Apogee Imaging Systems, Inc., Roseville, CA, USA). Dry intact seeds were vaccuum dried and gold coated (Sputter Coater SCD 050, Bal-Tec) before imaging with scanning electron microscope (JSM-6380LV; JEOL, Tokyo, Japan). Twenty days (after flowering) old pods of parental lines (JI64 and JI92) as well as RILs of F6 were fixed in 2% formaldehyde and stored in 4°C for latter observation of pod suture. Samples were cut on hand microtones at thickness of 100 μm, and the resulting segments were stained 1% phloroglucinol (Sigma, USA) in 12% HCl (Soukup, 2014).

### Liquid chromatography–electrospray ionization/mass spectrometry (LC/ESI-MS) analysis

Testa was separated from the rest of the seed, crushed, and extracted using mixture of acetone:water (70:30, v/v) with addition of 0.1% ascorbic acid to achieve efficient extraction of polyphenolic compounds in wide range of polarity and structural diversity (adapted from Amarowicz et al., 2009). 0.5 ml of extract was dried under a stream of nitrogen and solid residue was dissolved in 0.5 ml of methanol. The samples were then analyzed by ultra-performance liquid chromatograph Acquity UPLC I-Class coupled to high resolution tandem mass spectrometer Synapt G2-S with ion mobility separation capability (Waters, Milford, USA). Chromatographic column Raptor ARC-18 (100 × 2.1 mm, dp = 2.7 μm, Restek) and mobile phases (MP) A: water + 0.1% formic acid, B: acetonitrile + 0.1% formic acid was used for separation of components present in seed coat extracts. Flow rate of mobile phase 0.2 ml/min was applied. Electrospray was used as ion source. Spray voltage 2.5 kV in positive and 1.5 kV in negative ion mode, were used, respectively. Process of the LC/ESI-MS method optimization and detailed setup of mass spectrometer will be provided in Válková et al. (in preparation).

### Laser desorption/ionization–mass spectrometry (LDI-MS) analysis

Seeds of each genotype/line were mechanically disrupted and the seed coats were separated and pooled (four seeds per genotype). Description of the studied RILs is given in Table S1. Small pieces ~2 mm were fixed on MALDI plate using a common double sided adhesive tape. The samples were analyzed directly without application of a matrix. The prepared samples were analyzed using high resolution tandem mass spectrometer Synapt G2-S (Waters) equipped with vacuum MALDI ion source. For desorption/ionization a 350 nm 1 kHz Nd:YAG solid state laser was used. Details of LDI-MS setup and analytical parameters of hydroxylated fatty acids can be found in Cechová et al. (in preparation).

### Metabolite data treatment

The obtained LC/ESI-MS and LDI-MS data were processed by MarkerLynx XS a software extension of MassLynx platform (Waters). The processed data matrix, i.e., after extraction, normalization and alignment of retention times (in case of LC/ESI-MS data), m/z-values and intensities of signals, were transferred to Extended Statistics (XS) module, EZinfo (Umetrics, Malmo, Sweden), and studied by principal component analysis (PCA) and orthogonal projections to latent structures discriminant analysis (OPLS-DA). Both PCA and OPLS-DA were used for reduction of data dimensionality. OPLS-DA is a multivariate statistical method employing latent variable regression developed as an extension of more frequently used partial least squares method (Trygg and Wold, 2002). Coordinates of particular samples and RT_m/z pairs (or m/z-values in the case of LDI-MS data) in appropriate biplots and S-plots were used for evaluation of dormant and non-dormant genotypes mutual segregation and significance of detected signals of metabolites. The procedure was adopted and modified from Kučera et al. (2017). The most significant markers were further studied by targeted MS/MS experiments to reveal their identity (Cechová et al., in preparation; Válková et al., in preparation).

### RNA isolation

For Massive analysis of cDNA Ends (MACE) each sample of parental genotype was composed by several pooled developmental stages of seed coat (2, 3, 4 weeks and older) as it is unknown at which stage putative candidate genes are expressed. Seven selected RILs forming each of dormant resp. non-dormant bulk were previously (at F_6_ generation) and after mature seed harvest (of F_7_ generation used for RNA isolation) tested for germination behavior (Table S1). Seed coat were dissected under stereomicroscope, immediately frozen in liquid nitrogen and stored at −70°C until use. Frozen seed coats or dorsal and ventral sutures of pods were ground to a fine powder with liquid nitrogen using sterile mortars and pestles. Total RNA was isolated from seed coat (~100 mg) using the BioTeke Plant Total RNA Extraction Kit (China) or NucleoSpin RNA Plant kit (Macherey Nagel) for pods, according to the manufacturer's instructions. Yield/quantity and purity was determined by using NanoDrop 2000 spectrophotometer (Thermo Scientific) and diluted in DEPC-H_2_O to 100 ng/μl. Isolated RNAs were treated with DNase according to Baseline-ZERO™ DNase protocol (Epicenter). In case of parental genotypes (JI64, JI92, Cameor, and VIR320) four consecutive developmental stages of seeds (14–37 DPA) were taken each represented by 1.25 μg of total RNA. The RIL bulks were made of 1.425 μg of total RNA of each of seven lines. In case of RNA samples used for pod dehiscence study, two parental lines (JI64 and JI92) and two bulks of contrasting RILs (with dehiscent or indehiscent pods) were used. The bulk of dehiscent RILs was established from eight and bulk of indehiscent RILs from five lines using excised pod sutures of 10 and 20 days after flowering. Each of these four final samples contained ~1 μg of total RNA each. The integrity of the RNA samples was examined with an Agilent 2100 Bioanalyzer (Agilent Technologies, Palo Alto, USA).

### Massive analysis of cDNA ends (MACE)

MACE libraries were generated using GenXPro's MACE kit (GenXPro GmbH, Frankfurt, Germany) as described in Zawada et al. (2014). Briefly, cDNA from 5 μg of total RNA was randomly fragmented and biotinylated 3′ ends were captured after binding to a streptavidin matrix. A library ready for high-throughput sequencing was prepared using TrueQuant adapters included in the kit. The library consisted of 50–700 bp-long fragments derived from the 3′-end of the cDNAs. The 5′-ends of the libraries were sequenced on a HiSeq 2000 machine (Illumina) with 100 cycles to generate the MACE tags, each tag representing one single transcript molecule. In total, 6 cDNA libraries were prepared and sequenced for seed dormancy, while 4 libraries for parents and two contrasting bulks for pod dehiscence, each providing over 10 million reads (Table S2).

### Bioinformatics

After sequencing the reads are in 5′–3′ orientation. To remove PCR-bias, all duplicate reads detected by the TrueQuant technology were removed from the raw datasets. Low quality sequence-bases were removed by the software Cutadapt (https://github.com/marcelm/cutadapt/) and poly(A)-tails were clipped by an in-house python-script. The reads were aligned to reference sequences using Novoalign (http://www.novocraft.com). This tool maps reads to reference sequences depending on certain parameters (i.e., quality) and calculates thresholds for each assignment. The reference sequences consisted of all *Pisum* mRNA sequences from NCBI. We annotated these sequences to all Fabaceae proteins from Uniprot “http://www.uniprot.org/” by BLASTX to Swissprot (“sp|.,” good annotation) and afterwards to Trembl (“tr|…,” less good annotation) protein sequences. All reads that could not be mapped to Pisum mRNA sequences from NCBI were used for a *de novo* assembly to generate contigs denoted as “noHitAssembly_xxx” and annotated in the same way as the Pisum mRNA sequences from NCBI. Normalization and test for differential gene expression between the bulks were calculated using the DEGSeq R/Bioconductor package (Wang et al., 2010). Differential gene expression was quantified as the log_2_ ratio of the normalized values between two libraries (log_2_ FC). The *p*-value and correction for multiple testing with the Benjamini–Hochberg false discovery rate (FDR) were computed due to determining significance of gene expression differences in pairwise comparisons of libraries. Lists of Differentially Expressed Genes (DEGs) for three comparisons of contrasting phenotype (dormancy: wild × cultivated, RILs and their parents, dehiscence: RILs and their parents) were made based on combination of pairwise comparisons of log_2_ FC ratio of the normalized values (log_2_ FC > 2, log_2_ FC < −2) and FDR (FDR <0.01) between all libraries of these groups.

### GO and KEGG annotation

Gene Ontology (GO) enrichment analysis and normalized gene expression data were used to identify function and relationships of differentially expressed genes (Young et al., 2010). The results of the GO analysis were then exported into the Blast2go for the final annotations. The annotations provided the fragments with blast hit with the appropriate gene ontology terms which were classified into three categories: biological process (BP), cellular components (CC), and molecular function (MF). The DEGs were subjected for their presence in the different Kyoto Encyclopedia of Genes and Genomes (KEGG) pathways. The various enzyme activities and the DEGs involved in the KEGG pathways were revealed for each of the combinations.

### Genetic mapping of dehiscence specific SNPs-methodology

Transcripts containing SNP with at least five reads in both samples that are homozygous distributed in dehiscent vs. indehiscent RILs bulks with only one false allele read in 100 in either bulks were considered dehiscence specific. SNPs were discovered using Joint-SNV-Mix (Roth et al., 2012). The output given by Joint-SNV-Mix was furthermore processed by GenXPro in-house software to filter the SNPs. A minimum coverage of 10 bp was needed to be identified as an SNP. To identify the genomic localization of the SNP the surrounding region of the SNP was assigned per blastn to the genome of *M. truncatula* “JCVI.Medtr.v4.20130313” from http://jcvi.org/medicago. The “snpviewer” a webtool from the “http://tools.genxpro.net” was used to visualize the data.

### Real-time quantitative reverse transcription PCR

Gene-specific oligonucleotide primers were designed (Table S3) based on MACE consensus sequences using the FastPCR software (Kalendar et al., 2014). The expression of selected candidate genes was validated by quantitative real time PCR (qRT-PCR). RNA samples (treated with DNase) were reverse-transcribed with Oligo(dT)_15_ primer (Promega) in a two steps reaction in final volume 40 μl. The qRT-PCR analysis was run on the CFX96™ Real-Time Detection System (Bio-Rad) using the SensiFast SYBR® No-ROX kit (Bioline) or LightCycler® 480 SYBR Green I Master kit (Roche) in case of pod dehiscence study. Primers were designed using FastPCR or Oligo Primer Analysis Software (Molecular Biology Insights, USA) and produced amplicons ranging from 77 to 220 bp (Table S3). Every PCR reaction included 2 μl cDNA (1:10 diluted cDNA), 5 μl 2 × SensiFAST SYBR mix or LightCycler® 480 SYBR and 400 nM of each primer in final volume 10 μl. The expression was studied at two developmental stages in four contrasting parental genotypes (JI64, JI92, Cameor, and VIR320) and in case of dehiscence study also contrasting RILs with dehiscent or indehiscent pods. The conditions for PCR were: 95°C for 2 min; 45 cycles of 95°C for 10 s, 55°C for 30 s, and 72°C for 20 s; followed by a melting curve of 65–94°C (recovered every 0.5°C held for 0.5 s). The specificity of primers was confirmed by melting curve and gel analysis of products. Quantification of transcript level was determined by CXF Manager Software (Bio-Rad). Actin or β-tubulin gene was used as a reference to normalize relative quantification (Ferraro et al., 2014) using the comparative Ct (2^−ΔΔCt^) method. Changes in transcript were estimated as fold change relative to the expression in the genotype Cameor (younger stage) in case of seed dormancy study and genotype JI92 (younger stage) in case of pod dehiscence study.

## Results

### Seed coat mediated dormancy of wild pea seeds

#### Anatomical and germination differences between seed coat of wild and cultivated peas

**Figure 1:**
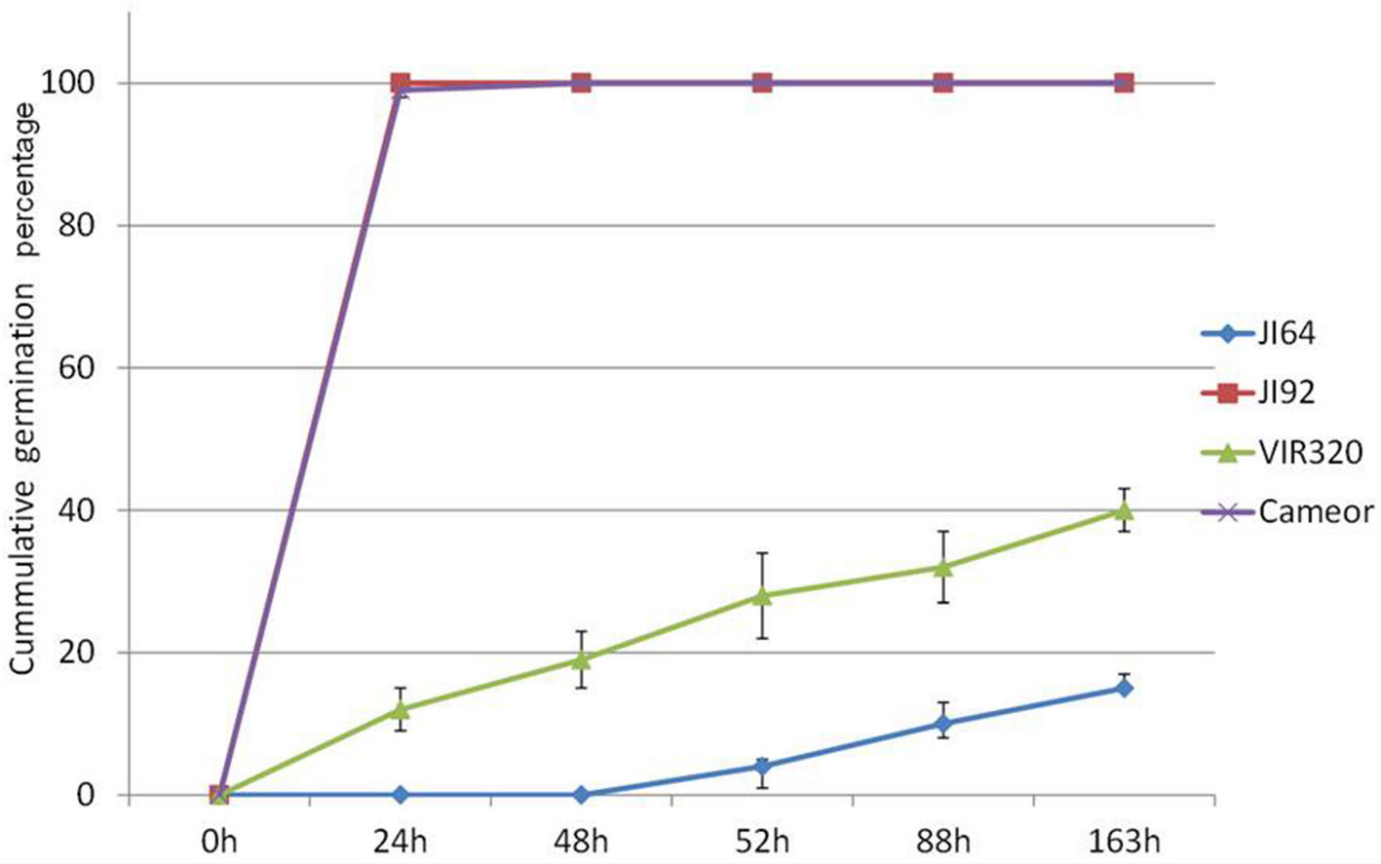
**Cumulative germination percentage of wild *P. elatius* (JI64, VIR320) and cultivated *P. sativum* (JI92 and cv. Cameor) seeds tested at 25°C over the period of 163 h**.

Wild pea seeds display high level of dormancy mediated by seed coat permeability. Two contrasting parental pairs of wild (*P. elatius*) JI64 and VIR320 and cultivated (*P. sativum*) cv. Cameor and JI92 peas were selected as they differ in testa pigmentation, thickness, and dormancy levels as well as pod dehiscence trait. Moreover, RILs were generated from cross of JI64 and JI92 to facilitate mapping. While cultivated pea seeds imbibe readily and germinate within 24 h (JI92, cv. Cameor), wild pea seeds remain highly dormant and imbibe and germinate at 8% (JI64) or 30% levels (VIR320) after 7 days (Figure 1). Mean germination time, Timson index, and Coefficient of Velocity are also very different between respective parental genotypes, 3.4 (MGT), 1 (TI), and 0.29 (CV) for JI92 while being 7, 0.008, and 0.14 for JI64, respectively. RILs displayed more variability in each of the respective measures (Figure S1), with wide range of percentage of germination (at 7 days) from 4 to 100%, 0.61 to 4.44 CV, 1 to 7 MGT, and 0 to 0.95 TI. All these parameters indicate complexity of imbibition and germination processes as none single is sufficient to fully describe any given line. As shown in Table S1, non-dormant bulk had on average 56% germination over 7 days period, 1.58 CV, 0.36 TI, and 68.02 MGT respective, contrast to dormant bulk lines having 22%, 1.38 CV, 0.28 TI, and 104.71 MGT, respectively. Similarly testa thickness was 107 vs. 135 μm, respectively.

**Figure 2:**
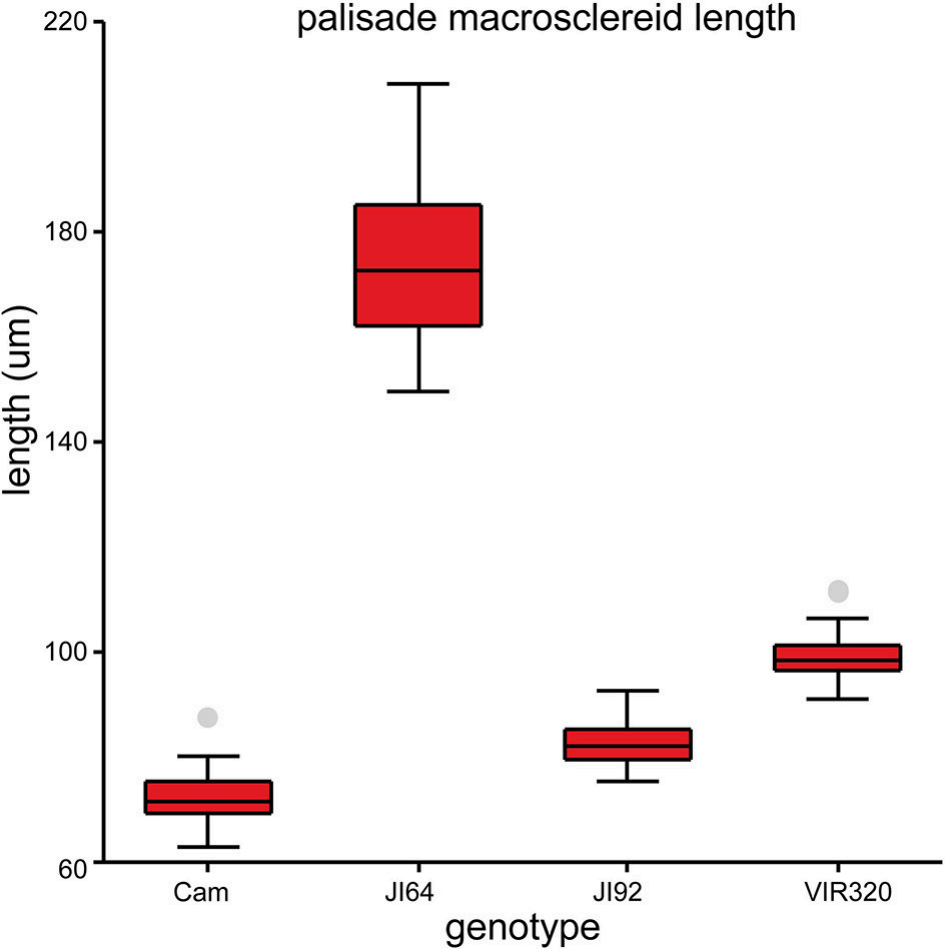
**Length of the seed coat palisade cells of selected pea genotypes (Cameor, JI92, JI64, and VIR320)**. Box plot of median with 25th and 75th percentile, whiskers are 5th and 95th percentile; *n* = 75; each genotype is different from the other (ANOVA *p* < 0.001).

**Figure 3:**
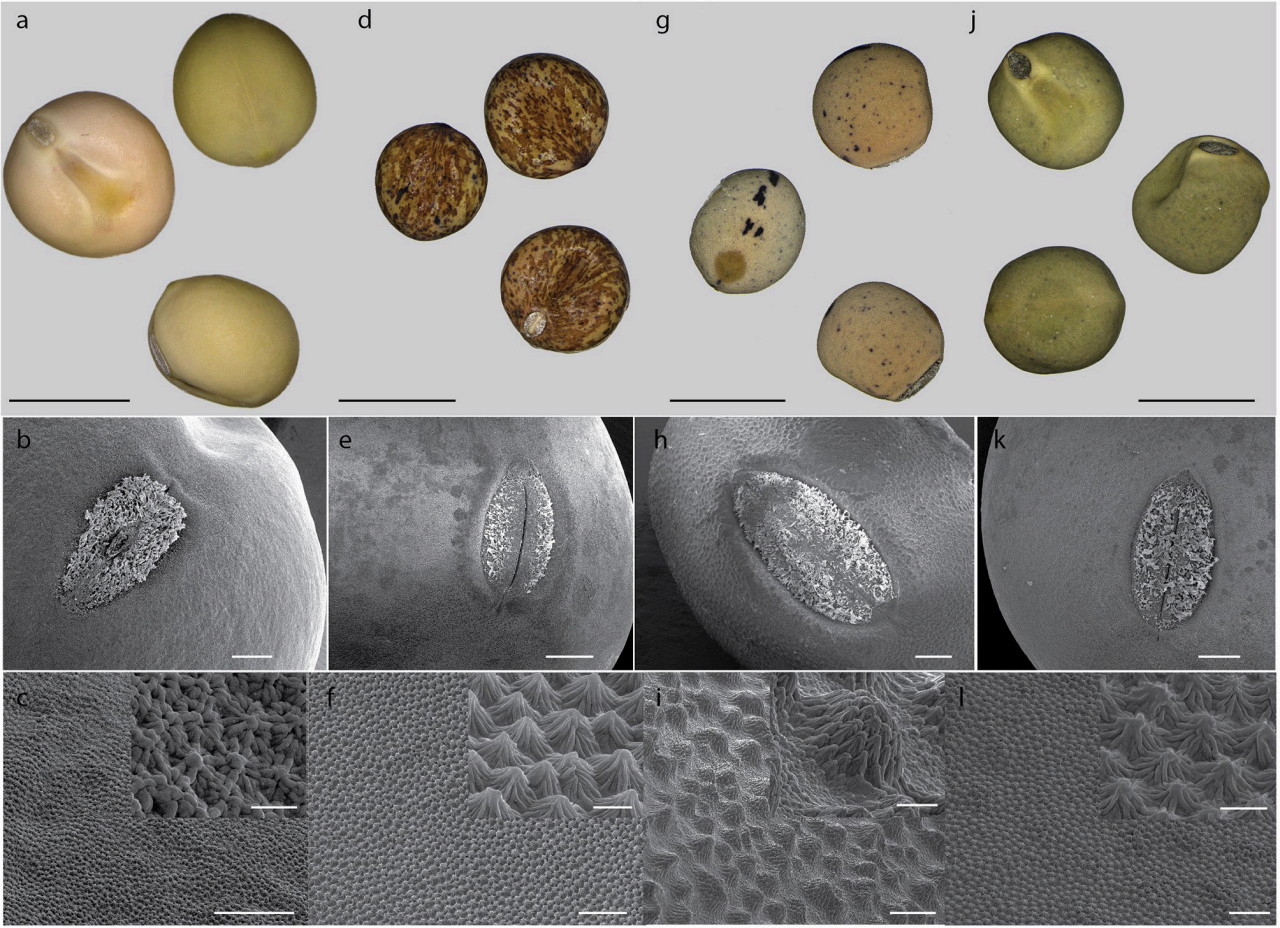
**Seed coat surface of selected pea genotypes**. Pigmentation of seed coat: Cameor **(a)**, JI92 **(d)**, JI64 **(g)**, and VIR320 **(j)**; scale bar = 5 mm. Surface texture (SEM): Cameor **(b,c)**, JI92 **(e,f)**, JI64 **(h,i)**, and VIR320 **(k,l)** overall view, scale bar = 500 μm; details of extrahilar region **(c,f,i,l)** with inlay of high magnification, scale bars = 100 μm **(c,i)** and 50 μm **(k,l)**, inlay scale bars = 10 μm **(c)** 20 μm **(l)**.

**Figure 4:**
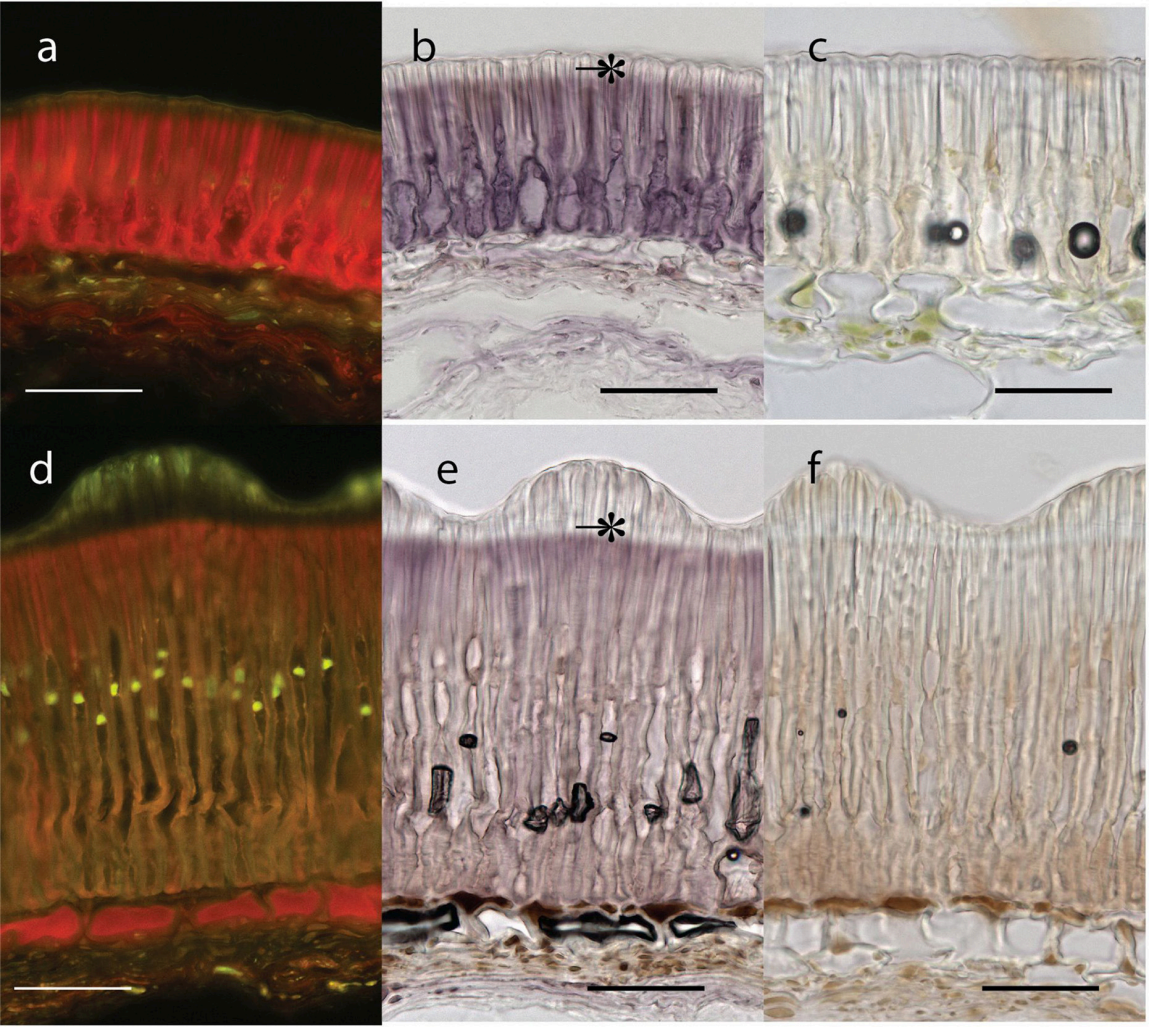
**Seed coat transverse sections from extrahilar region stained with DMACA for proanthocyanidins polyphenolics** (bar = 50 μm): Upper row—JI92, Lower row—JI64. Blue excited red fluorescence of DMACA **(a,d)** and violet coloration in bright field **(b,e)**. Similar sections as in **(c,f)** but stained with DMACA after mild alkaline hydrolysis; scale bars = 50 μm. ^*^, Light line.

Testa thickness was analyzed by micrometric, light microscopy or SEM measurements. Especially JI64 and JI92 differ substantially in palisade cells length, which contributes to overall testa thickness (Figure 2). Dormant genotype JI64 has significantly thicker testa, which might contribute to the water impermeability of seed coat of dormant pea genotypes. There are considerable differences in surface pigmentation and texture of individual lines (Figures 3a,d,g,j). While Cameor is not pigmented visible pigmentation is present in other genotypes with different intensity and localization. The texture of surface is variable among genotypes, particularly in details of macrosclereid tips arrangement defining the surface shape being covered by thin cuticle (Figures 3b,c,e,f,h,i,k,l and Figure S2). The most obvious is gritty surface of JI64 which was absent in all the other genotypes included in this study. The continuity of surface (cuticle integrity) was interrupted locally by minor fissures in all genotypes. Large fissures developed in seed coat of the non-dormant genotypes Cameor and JI92, mostly in the hilar and strophiolar region later during the imbibition (data not presented). The cytological arrangement of seed coat of tested genotypes varies particularly in macrosclereids. The surface is covered with thin cuticle (Figure S2) which copies the outer extremities of palisade macrosclereids. Based on light microscopy we did not see any apparent difference in cuticle properties among genotypes as revealed by autofluorescence or Sudan staining (Figure S2). Interestingly, cuticle is not the only lipidic material localized close to the seed coat surface. Non-cuticular lipidic extracellular material was present also in the very tips of the macrosclereids (Figures S2e–h) containing autofluorescent material (Figures S2a–d). The analysis of seed coat surface was complemented with histochemical analysis of selected compounds of cell wall. Metachromatic staining with toluidine blue of non-dormant genotype Cameor exhibited high level of polyanionic cell wall components, while in contrary non-dormant, but well-pigmented JI92 showed lower abundance of polyanionic compounds similarly to dormant genotypes where metachromasy was mostly limited to macrosclereid tips bellow the cuticle (see Figures S2a–d). Staining with Alcian blue further supports such conclusion (data not shown). Interestingly, metachromatic staining was enhanced with 3% acetic acid pretreatment (Figures S3e–h). Clear connection between presence of tannins and toluidine blue stainability is well-documented in JI92, where deeper staining is present out of pigmented spots (proanthocyanidin positive; Figure S3f). The presence of proanthocyanidin within cell walls of macrosclereids was not detected in non-dormant genotype Cameor but was obvious in JI92 as well as in the dormant genotypes (Figure 4) using both HCl-Vanilin and DMACA tests. Condensed tannin presence was never recorded within the light line (Figure 4) of macrosclereids of any genotype. Macrosclereids of dormant type genotypes seems to be enriched with proanthocyanidins in the cell walls of the entire macrosclereids up to the light line. Seed coat of JI92 is highly enriched with proanthocyanidins only in the dark pigmented spots. Interestingly, the abundance of condensed tannins negatively correlates with toluidine blue stainability and sirofluor staining of cell walls indicating tannin linkages to other compounds within the cell wall. Detected tannins are not extractable with ethanol, or 1M HCl. Alkaline hydrolysis of cell wall bound tannins by 1M sodium hydroxide resulted in loss of tannins stainability (Figures 4c,f). There was intense aniline blue staining of macrosclereids in all genotypes, particularly in the light line of macrosclereids (Figures S2i–l) and their outer part composed in majority of the secondary cell walls. The strongest signal was observed in dormant genotypes, especially in the light line. However, the signal for callose specific antibody did not correspond with aniline blue fluorophore and in general was found rather week and discretely localized (Figure S2i—inlay). Phloroglucinol staining indicative of lignin provided no response in non-dormant nor in dormant pea genotypes indicating the absence of significant amount of lignins in the testa of analyzed pea genotypes.

#### Chemical analysis of seed coat composition

**Figure 5:**
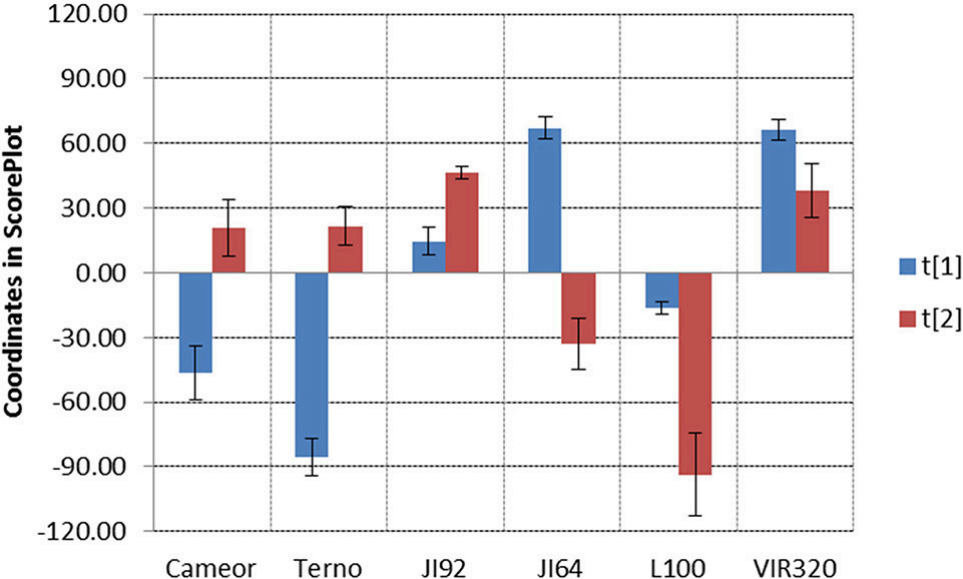
**Score Plot of LC/ESI-MS data of dormant and non-dormant pea genotypes. Data filtration threshold 1000 counts in MS spectrum; replicated analyses, three repetitions of each genotype, level of significance α = 0.05, UV scaling, no transformation**.

**Figure 6:**
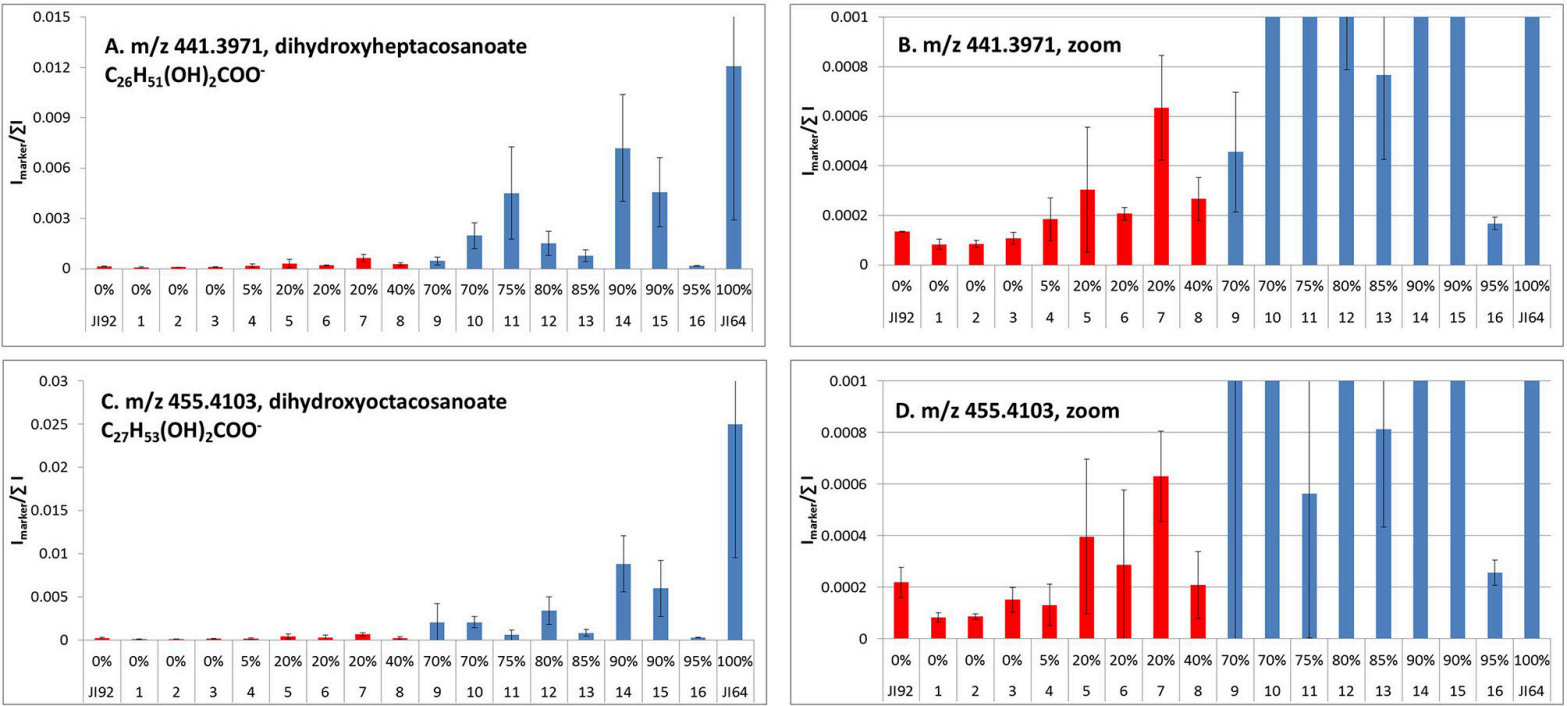
**LDI-MS analysis of dihydroxyheptacosanoate (A)** and dihydroxyoctacosanoate **(C)** in seed coats of contrasting parents (JI64, JI92) and selected RILs (intensity of signals at particular m/z-values normalized to sum of all signals in mass spectrum; red, non-dormant; blue, dormant lines; error bars reflect the standard deviation of three replicated measurements, α = 0.05); **(B,D)**, zoomed graphs.

Detection of metabolites present in seed coat related to dormancy was based on comparison of LC/ESI-MS and LDI-MS data of dormant and non-dormant pea genotypes using principal component analysis and orthogonal projection to latent structures. Figure 5 reflects the differences in coordinates of particular genotype samples in corresponding Score plot obtained by Principal Component Analysis of LC/ESI-MS data. Although, individual coordinates do not exhibit statistically significant differences among all the genotypes (e.g., t[2] coordinates of Cameor, Terno, and VIR320), location of each genotypes given by combination of both coordinates provided resolution among particular genotypes. The differences in the coordinates (mutual orientations and values) clearly show the separation of dormant (i.e., L100, JI64, and VIR320) from non-dormant genotypes (i.e., Terno, Cameor, and JI 92) using the acetone:water extract. Separation of L100 and JI64 from non-dormant genotypes is much more significant compared to the separation of VIR 320. This can be explained by possible semi-domesticated status of this genotype. Based on the achieved separation of dormant and non-dormant genotypes by unsupervised Principal Component Analysis (PCA), supervised Orthogonal Projection to Latent Structures (OPLS-DA) was used to find signals mostly responsible for the chemical differences in dormant and non-dormant samples. Those signals (m/z-values of markers with increased intensity in dormant genotypes compared to non-dormant ones) were studied in detail by targeted tandem mass spectrometry (study of their fragmentation after collision induced dissociation in collision cell of mass spectrometer). Details of analytical interpretation can be found in Válková et al. (in preparation). Attention was especially focused on the chemical differences between morphologically the most similar pair of genotypes, i.e., JI 64 and JI 92. Combination of information about retention time, exact mass measurement and fragmentation revealed the identity of the most significant dormancy markers found in acetone-water extracts—dimer and trimer of gallocatechin (m/z 611.1387 and 915.1945, deviation of measured from theoretical m/z-value of parent ion, dtm, –0.8 and –3.3 mDa), respectively, quercetin-3-rhamnoside (m/z 449.1045, dtm –3.3 mDa) and myricetin-3-rhamnoside (m/z 465.1112, dtm 7.9 mDa). Analogously, the chemical differences between dormant JI64 and JI92 genotypes were studied by laser desorption-ionization mass spectrometry (LDI-MS). Measurement in negative ion mode in combination with PCA a OPLS-DA revealed marked differences in the profile of particular hydroxylated long chain fatty acids [i.e., m/z 411.3865, hydroxyhexacosanoate (dtm 2.7 mDa); m/z 425.3927, hydroxyheptacosanoate (dtm −6.8 mDa); m/z 427.3875, dihydroxyhexacosanoate (8.8 mDa); m/z 437.4033, hydroxyoctacosanoate (dtm 3.8 mDa); m/z 441.3973, dihydroxyheptacosanoate (2.9 mDa) and m/z 455.4180, dihydroxyoctacosanoate (dtm 8.0 mDa)]. Two orders of magnitude higher normalized signals of dihydroxyheptacosanoate and dihydroxyoctacosanoate were measured in JI 64 compared to JI 92, i.e., (1.21 ± 0.92).10^−2^ and (2.50 ± 1.54).10^−2^ vs. (1.34 ± 0.02).10^−4^ and (2.19 ± 0.58).10^−4^, respectively. Figure 6 shows the normalized signals of both dihydroxylated long chain fatty acids in seed coats of JI64, JI92, and RILs with respect to dormancy. The majority of dormant RILs exhibit higher content of those fatty acids compared to non-dormant ones.

#### Seed coat transcriptome differences between wild and cultivated pea

**Figure 7:**
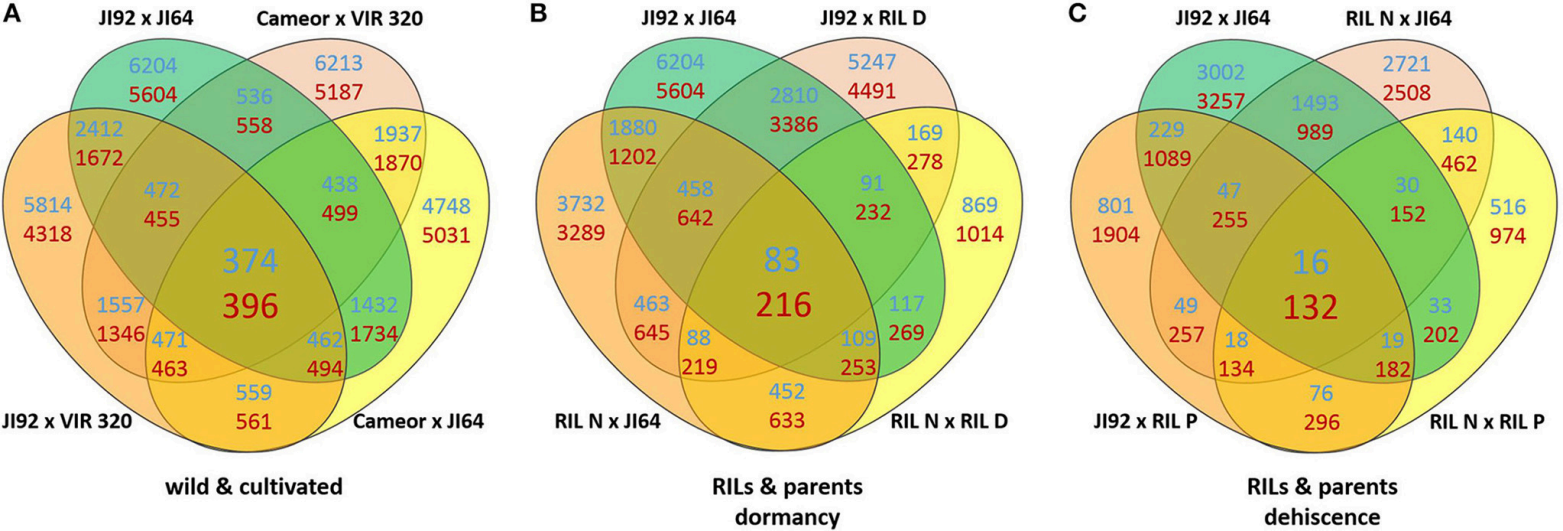
**Venn diagrams of differentially expressed genes (DEGs criteria log_2_ FC ≥ 2 and FDR ≥ 0.01) between seed coat (A)** of the studied wild and cultivated genotypes, **(B)** and JI64, JI92 parents and resulting RILs and **(C)** between pod sutures of JI64, JI92 parents and resulting RILs. Blue counts are upregulated and red counts are downregulated in cultivated genotypes.

In order to understand the mechanism of testa mediated dormancy we selected seed coat derived from two contrasting parental pairs and two bulks of RILs differing in dormancy level to extract RNA and to identify candidate genes using the next-generation sequencing method of Massive Amplification of cDNA Ends (MACE). The isolation of RNA from wild pea seed coat tissue proved to be very difficult. It is likely that high due to content of free metabolites (oligosaccharides and proanthocyanidins). We assessed the expression patterns in domesticated (cv. Cameor and JI92 landrace) vs. wild *P. elatius* (JI64, VIR320) pea. Each sample has yielded between 8 and 15 million clean reads (Table S2). Bioinformatics analysis resulted in identification of 144,000 transcripts (e.g., MACE annotated fragments) expressed in immature seed coat tissue. We have used stringent values of false discovery rate (FDR) ≤ 0.01 and fold-change (log_2_ FC) ≥2 as a threshold to identify the significant differences in the gene expression. Applying these criteria, a total of 10,132–11,808 transcripts were found differentially expressed between cultivated (JI92, Cameor) and wild (JI64, VIR320) parents (Table S2). Of these 770 were differentially expressed between all wild vs. cultivated genotypes, of these 374 were up-regulated in cultivated genotypes, and 396 down-regulated (Figure 7A, Table S4), when annotated to pea transcriptome, respectively.

**Figure 8:**
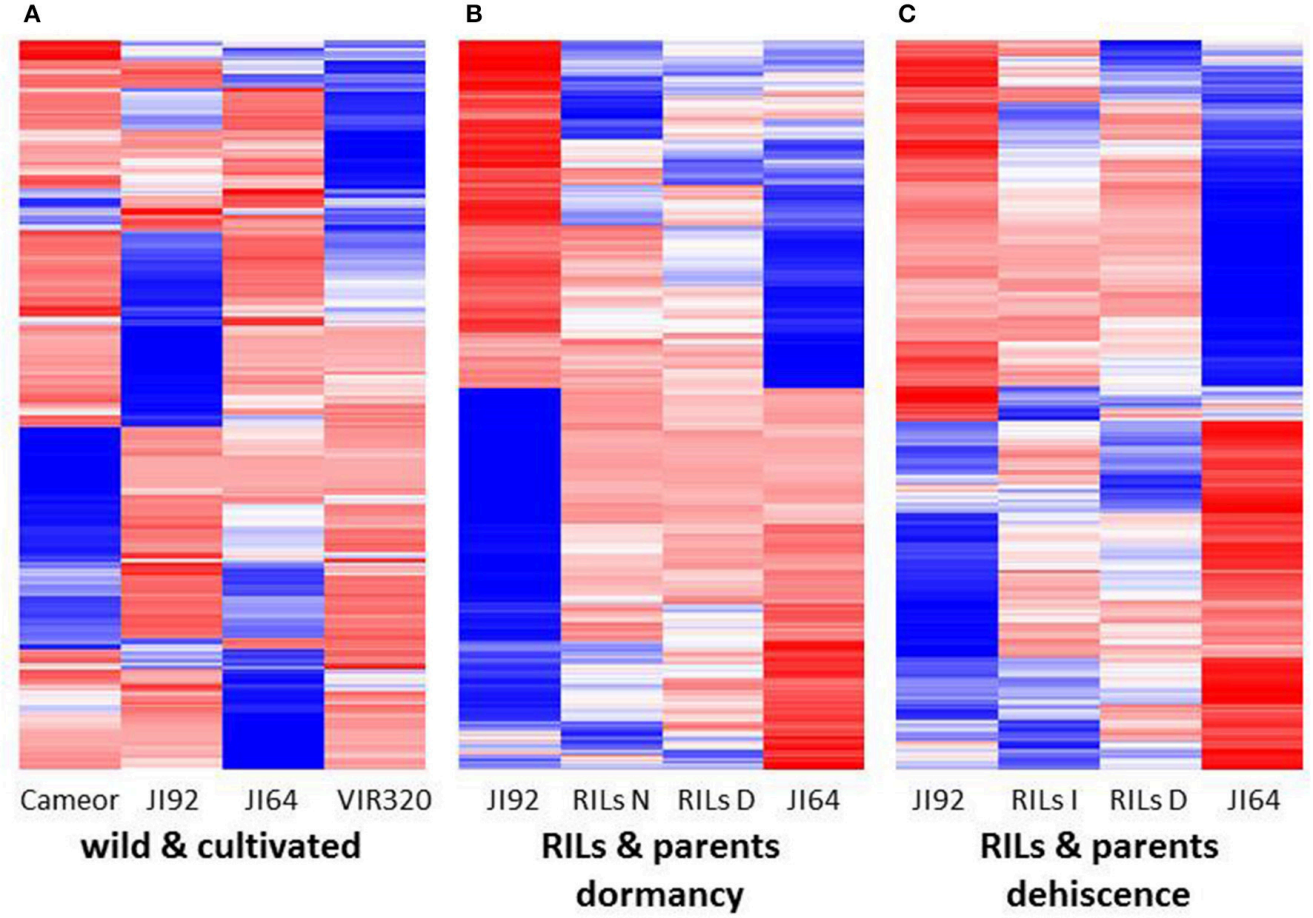
**The heat maps (A)** of 1,000 genes with the highest variance among normalized expression values for cultivated and wild pea samples, **(B)** of 11,808 DEGs for dormancy, and **(C)** of 6,259 DEGs for dehiscence between RIL's parents samples in MACE experiments.

A heat-map of 1,000 genes with the highest variance among normalized expression between cultivated and wild pea samples (Figure 8A) further illustrates differences between domesticated and wild pea seed coat expressed genes. This comparison shows the differences between respective pairs (cultivated vs. wild). In addition to contrasting parental genotypes, two bulks of seed coats at several pooled developmental stages of seven dormant and seven non-dormant RILs (Table S1) were analyzed. The bulks were included to minimalize identification of genes associated with respective genetic background rather than dormancy trait. This is clearly shown when DEGs are compared between parents and RIL bulks (having largest number of specific DEGs between parents e.g., 6,204 up-/5,604 down-regulated transcripts, while only 869 and 1,014 down-regulated transcripts in RIL non-dormant vs. dormant bulks. In case of gene expression profile of dormancy and non-dormancy RILs and their parents, 299 DEGs were found. When comparison included JI64 and JI92 parents and two respective RIL bulks, there were 83 up- and 216 down-regulated genes (Figure 7B). In order to visualize the expression pattern of RILs and theirs parents, heatmaps were constructed for 11,808 genes from libraries of dormancy and 6,259 genes from libraries of dehiscence (Figure 8B).

#### Verification of differentially expressed genes during seed coat development

**Figure 9:**
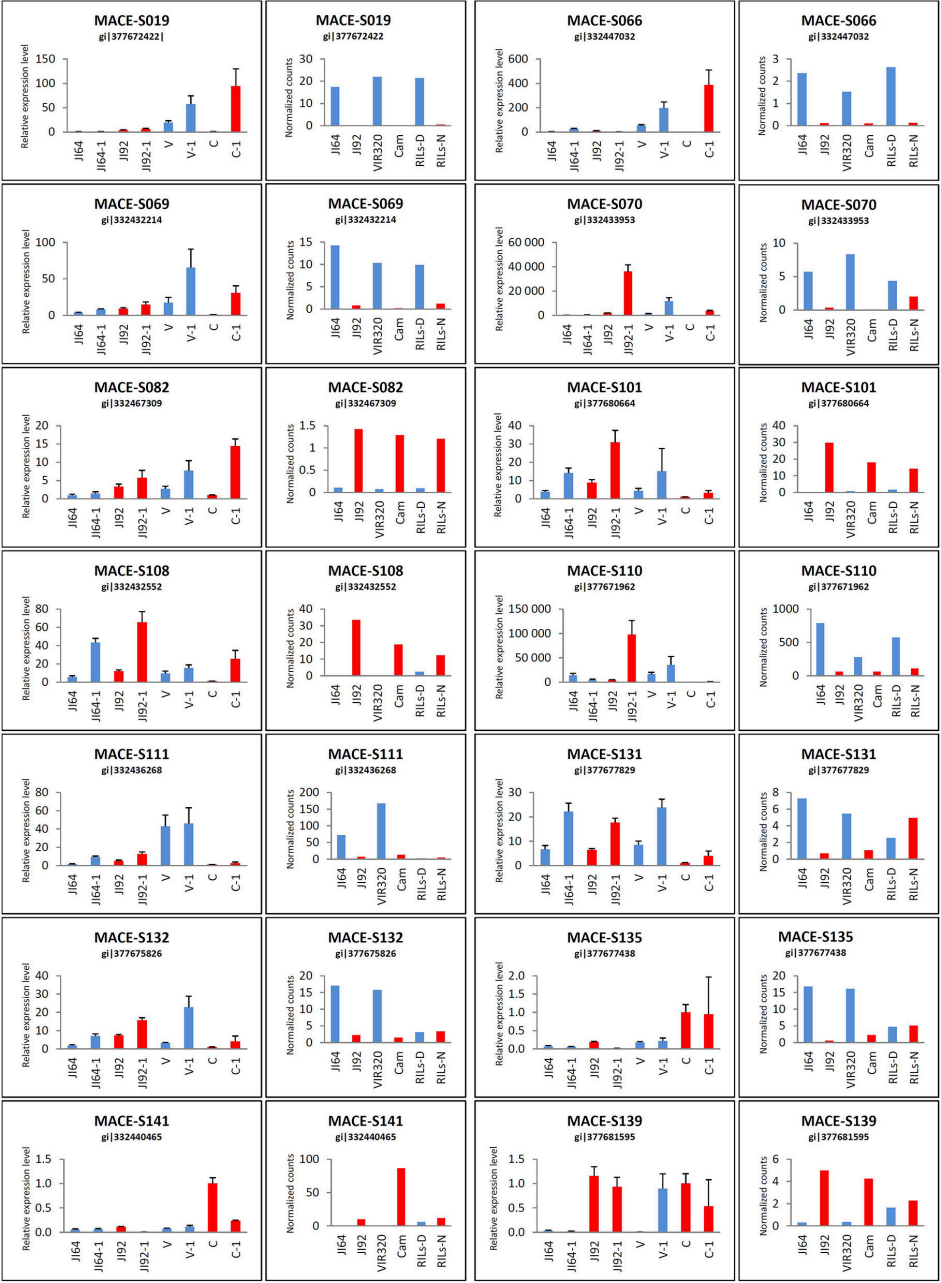
**qRT-PCR results of selected candidate genes in comparison with MACE analysis**. qRT-PCR for four genotypes in two stages: JI64, JI92, V (VIR320), and C (Cameor)—younger stage; JI64-1, JI92-1, V-1, C-1—older stage and MACE for JI64, JI92, VIR320, Cam, RILs-D (dormant), and RILs-N (non-dormant).

We selected DEGs based on expression level difference and gene annotation (Table S4), regarded as candidate genes for seed coat mediated dormancy in two possible directions of evolutionary changes (i.e., up- or down-regulated in the domesticated pea compared to its wild progenitor). To validate MACE results, expression levels of 14 selected DEGs was analyzed by qRT-PCR. According to MACE results, the selected genes comprised of five up-regulated genes (direction dormant to non-dormant): porin (MACE-S082), NADPH-cytochrome P450 reductase (MACE-S101), peroxisomal membrane PEX14-like protein (MACE-S108), UDP-glucose flavonoid 3-O-glucosyltransferase (MACE-S139), xyloglucan:xyloglucosyl transferase (MACE-S141), and 9 down-regulated genes: NADH-cytochrome b5 reductase (MACE-S019), divergent CRAL/TRIO domain protein (MACE-S066), 1-deoxy-D-xylulose, 5-phosphate, reductoisomerase (MACE-S069), probable aldo-keto reductase 1 (MACE-S070), cupin RmlC-type (MACE-S110), heavy metal transport/detoxification protein (MACE-S111), 4-coumarate CoA ligase (MACE-S131), cytochrome P450 monooxygenase CYP97A10 (MACE-S132), β-amyrin synthase (MACE-S135). Of these tested 14 genes, 4 genes (MACE-S082, MACE-S108, MACE-S131, and MACE-S139) were in agreement in all four genotypes with data obtained with MACE method (Figure 9). Relative expression level by MACE-S019 and MACE-S135 was completely contrary to MACE results, where higher values were in wild dormant genotypes. The qRT-PCR expression pattern of MACE-S066 and MACE-S101 were different in two genotypes (Cameor and VIR320) compared to MACE data, with MACE-S066 higher expressed in wild dormant (VIR320) and MACE-S101 higher expressed in cultivated non-dormant genotype (Cameor). Discrepancy between qRT-PCR and MACE methods in JI64 and JI92 was found in case of MACE-S069, MACE-S070, MACE-S110, MACE-S111, MACE-S132, and MACE-S141.

#### Enrichment analysis of DEGs functional classes between wild and domesticated pea seed coats

In order to investigate transcriptome changes in seed coat associated with evolution under domestication, we assessed the expression patterns of the DEGs in domesticated (cv. Cameor and JI92 landrace) vs. wild *P. elatius* (JI64, VIR320) pea. We identified 770 DEGs (583 respectively, when ambiguous are removed) is seed coat between wild and domesticated peas. Due to the absence of complete pea genome and likely specificity of seed coat tissue, we could annotate 66% of MACE fragments. Moreover, between 36 and 41% produced ambiguous assignment (Table S2). For DEGs sequences assigned to GO terms, we observed differences within all three compounds: cellular components, molecular function, and biological process. Several GO groups were found differently expressed. In GO enrichment of DEGs between wild and cultivated the most interesting results belongs to Molecular function group (Table S5). The most DEGs were found in phenylpropanoid (17 genes in KEGG pathway) and flavonoid (11 genes) biosynthetic pathways (Figures S4, S5). These included O-hydroxycinnamoyltransferase (EC:2.3.1.133), dehydrogenase (EC:1.1.1.195), O-methyltransferase (EC:2.1.1.68), gentiobiase (EC:3.2.1.21), lactoperoxidase (EC:1.11.1.7), ligase (EC:6.2.1.12), reductase (EC:1.2.1.44), and 4-monooxygenase (EC:1.14.13.11) or synthase (EC:2.3.1.74), reductase (EC:1.3.1.77), O-hydroxycinnamoyltransferase (EC:2.3.1.133), isomerase (EC:5.5.1.6), 3′-monooxygenase (EC:1.14.13.21), 4-monooxygenase (EC:1.14.13.11) genes (Table S5), respectively. Enzyme 4-coumarate CoA ligase (MACE-S131) catalyzes conversion of 4-coumarate and other derivatives to corresponding esters serving to generate precursors for formation lignin, suberin, flavonoids. In general, main differences in gene expression was detected between enzymes that played important role in secondary metabolites biosynthesis. Different levels of expression were observed for the cellulose synthase enzyme (EC:2.4.1.12) and two enzymes of pectin metabolism pectate lyase (EC:4.2.2.2) and pectin methylesterase (EC:3.1.1.11), which may interfere together with enzymes from the phenylpropanoid and lignin biosynthesis to the structural composition of cell wall. Enzyme 3-hydroxyacyl-CoA (EC:1.1.1.35) dehydrogenase that belongs to fatty acid elongation pathways is also up-regulated in dormant (wild) genotypes. This enzyme participating in of fatty acid biosynthesis showed changes in expression between wild and cultivated genotypes (Figure 6). Out of 548 DEGs, 307 (56%) were assigned to GO-term groups, including 171 (56%) down-regulated and 136 (44%) up-regulated in the domesticated pea seed coat compared to wild samples. As shown in Table S5, the known DEGs were mainly classified into 40 functional categories and involved in 19 biological processes. The results showed that these DEGs mainly distributed in plasma membranes and nucleus after genes expression, and participated in the biological process of biosynthetic process (60 genes, 12%), metabolism (183 genes, 36%), regulation of transcription (3 genes, 0.5%), transporting (17 genes, 3%), stress response (16 genes, 3%), cell division and differentiation (180 genes, 35%), localization (30 genes, 6%), establishment of localization (28 genes, 6%), lignin synthesis and so on. Through comparative analysis, the two most abundant sub-classes were biosynthesis processes and metabolic processes. The KEGG pathway analysis showed the presence of many genes PsCam051542, PsCam037704, PsCam043296, PsCam000856, PsCam038256, PsCam049689, PsCam016941, PsCam049689, PsCam050533 involved phenylpropanoid biosynthesis. Similarly, PsCam049689, PsCam038227, PsCam005153, and PsCam050665 were found to be associated with flavonoid biosynthesis (Table S5).

### Pea pod dehiscence

The structure of pea pericarp follows the common arrangement in *Fabaceae*. The exocarp consists of thick walled epidermis, the relatively thick mesocarp is arranged in several layers of parenchyma and endocarp composed of lignified sclerenchyma on inside of which is thin-walled epidermis. Both exocarp and mesocarp are rich in pectins, as indicated with metachromatic staining of toluidine blue (**Figure 11**).

#### Differentially expressed genes between dehiscent and indehiscent pods

**Figure 10:**
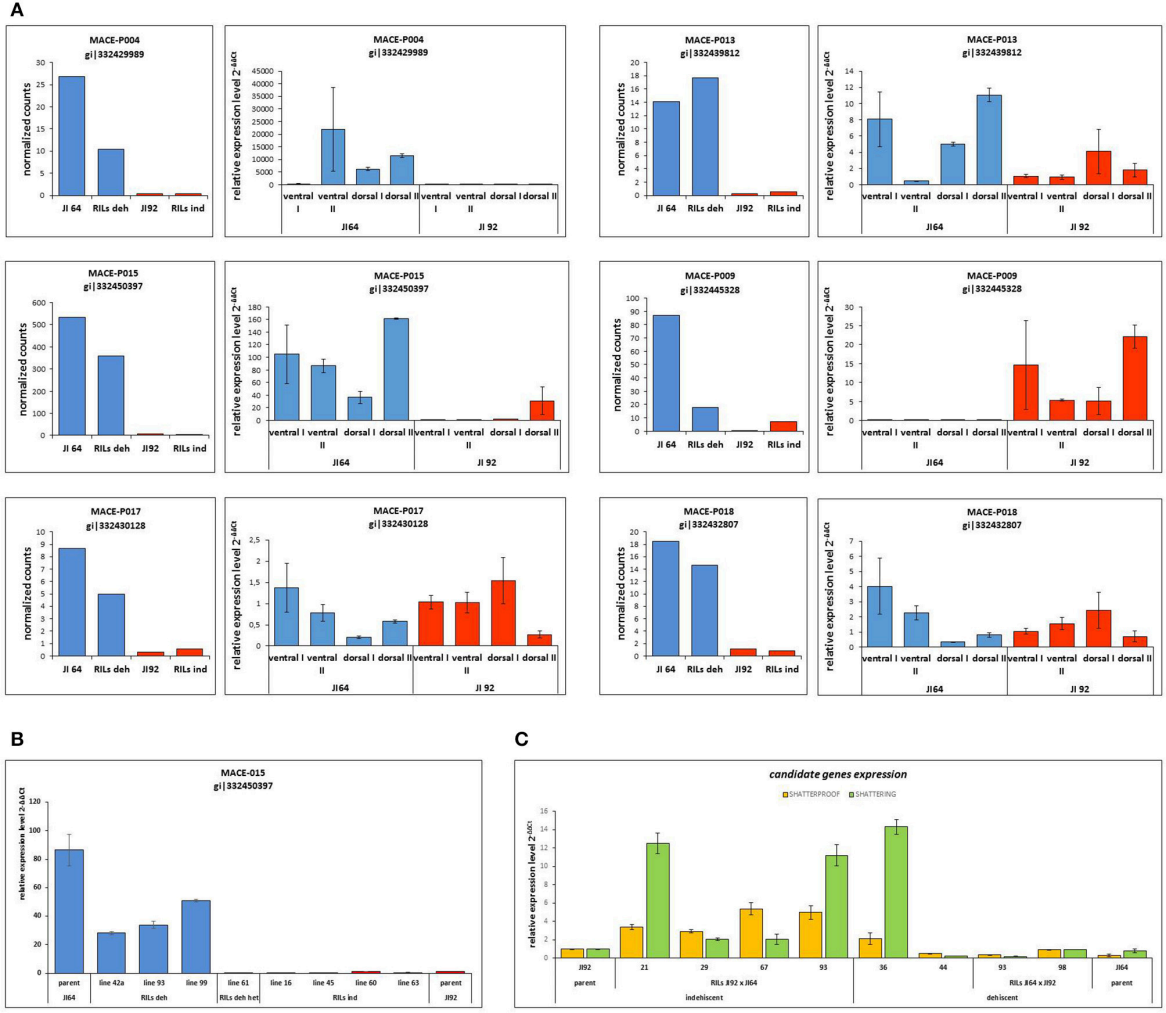
**qRT-PCR validation of selected DEGs detected to be associated with pod dehiscence. (A)** MACE results in comparison with qRT-PCR of selected candidate genes. MACE-P004, MACE-P013, and MACE-P015 shows the same trend of over/down expression between MACE results of parental lines and RILs (JI 64—dehiscent pod, bulk of dehiscent RILs, JI 92—indehiscent pod, bulk of indehiscent RILs) and qRT-PCR of cDNA of parental lines (ventral or dorsal pod suture in two stages: I—younger stage, II—older stage). MACE-P009 showed the opposite results and MACE-P017 and MACE-P0018 without any trend of down or over expression in qRT-PCR. **(B)** qRT-PCR results of contrasting RILs (dehiscent/indehiscent pod) in comparison with parental lines of candidate gene MACE-P015. **(C)** qRT-PCR results of contrasting RILs (dehiscent/indehiscent pod) in comparison with parental lines of candidate genes SHATTERPROOF and SHATTERING.

**Figure 11:**
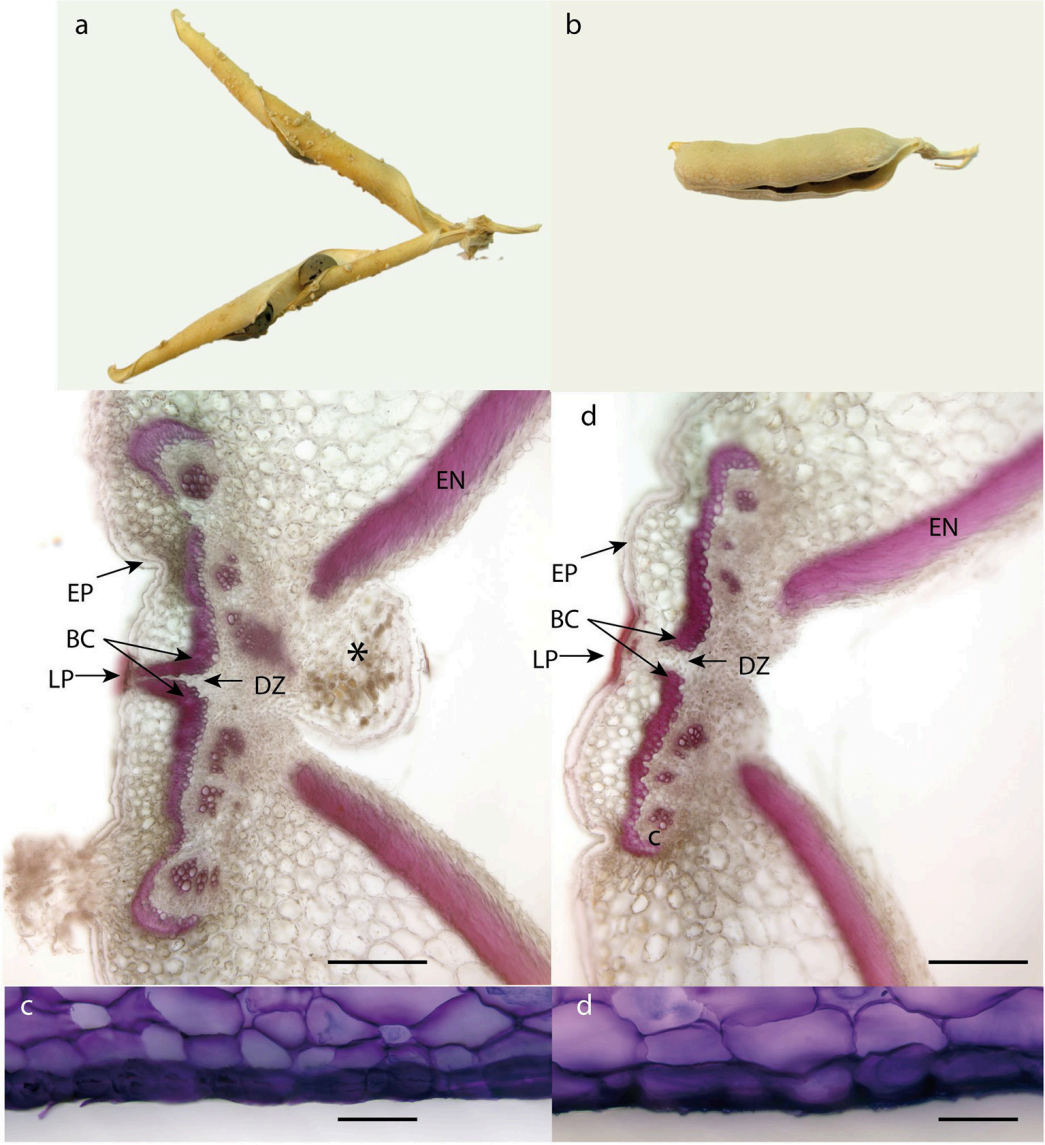
**Dorsal side of pea dehiscent and indehiscent pod. (a)** Mature pod of dehiscent line (JI 64). **(b)** Mature pod of indehiscent line (JI 92). **(c,d)** Sections of dorsal side of dehiscent and indehiscent pea pod stained with fluoroglucinol: ^*^, funikulus; BC, bundle cap; DZ, dehiscence zone; EN, endocarp, including inner sclerenchyma; EX, exocarp; EP, epidermis; DZ, dehiscence zone; LP, lignified epidermal plate.

In the case of pea pod dehiscence, we used MACE methodology to find differences in expression profiles between JI92 (domesticated, indehiscent pod) and JI64 (wild, dehiscent pod) and between two bulks of contrasting RILs. For each sample we used bulk of three developmental stages (2, 4 weeks and older) of dissected pod suture tissue (Table S2). Across all dehiscent and indehiscent libraries 148 DEGs were found (Figure 7C). Of these, 132 DEGs were down-regulated and 16 were up-regulated in indehiscent libraries. For gene expression analysis via qRT-PCR we selected 20 gene candidates with the most different expression in MACE analysis between contrasting lines (dehiscent and indehiscent pod). Nineteen of them were recognized by MACE as down expressed (with lower expression in domesticated indehiscent genotype) and one expressed gene candidate. In addition we tested also five other gene candidates (transcription factors) reported to be responsible for pod dehiscence in other plant species (bHLH Basic helix-loop-helix proteins *INDEHISCENT, SPATULA, SHATTERPROOF*, Basic Leucine Zipper Domain genes *bZIP* and *SHATTERING*; Ferrándiz et al., 2000; Girin et al., 2011; Dong et al., 2014). For these experiments we used RNA from pod suture tissue of parental line JI92 (domesticated, indehiscent pod) and JI64 (wild, dehiscent pod) as well as of eight contrasting RILs. As a result we detected over expression in indehiscent lines of two gene candidates (*SHATTERING* and *SHATTERPROOF*; Figure 10C) and the rest of tested genes (*INDEHISCENT, SPATULA, bZIP*) did not show differential expression. In the second experiment we tested gene expression of 20 gene candidates derived from MACE analysis. In this case, we tested parental lines JI64 and JI92 only. We used RNA from dorsal and ventral pod suture tissue of three developmental stages (10, 15, and 20 days after flowering). In this case we detected three genes (MACE-P004, MACE-P013, MACE-P015) corresponding to the down/over expression as in MACE results (Figure 10A). In *M. truncatula* genome homologs genes are: MACE-P004 transmembrane protein, putative (Medtr3g016200); MACE-P013 NADP-dependent malate dehydrogenase (Medtr1g090730), and MACE-P015 peptidoglycan-binding domain protein (Medtr2g079050). In one case (MACE-P009) qRT-PCR showed the opposite results than in MACE (Figure 10A). *Medicago* homolog of MACE-P009 gene is cathepsin B-like cysteine protease (Medtr7g111060). The rest of candidate genes for pod dehiscence generated by MACE were tested by qRT-PCR, namely: glycosyltransferase family 92 protein (MACE-P001, Medtr2g437660); serine carboxypeptidase-like protein (MACE-P002, Medtr3g434850); KDEL-tailed cysteine endopeptidase CEP1 (MACE-P003, Medtr3g075390); 60S ribosomal protein L3B (MACE-P005, Medtr1g098540); dormancy/auxin associated protein (MACE-P006, Medtr7g112860); enoyl-CoA hydratase 2, peroxisomal protein (MACE-P007, Medtr3g115040); disease-resistance response protein (MACE-P008, Medtr2g035150); GASA/GAST/Snakin (MACE-P010, Medtr1g025220); dormancy/auxin associated protein (MACE-P011, Medtr7g112860); TCP family transcription factor (MACE-P012, Medtr2g090960); LL-diaminopimelate aminotransferase (MACE-P014, Medtr2g008430); zinc finger A20 and AN1 domain stress-associated protein (MACE-P016, Medtr2g098160); auxin-responsive AUX/IAA family protein (MACE-P017, Medtr1g080860); huntingtin-interacting K-like protein (MACE-P018, Medtr2g034010); transmembrane-like protein (MACE-P019, Medtr2g038550) and vacuolar processing enzyme (MACE-P020, Medtr4g101730). In the third experiment, we tested only candidates which showed differences in expression in both previous experiments using eight RILs with clearly determined phenotype (dehiscent or indehiscent pods). Four RILs were phenotypically dehiscent and four indehiscent. In this case, we used cDNA derived from the mixture of dorsal and ventral pod sutures tissue (Figure 11) for analysis. Finally, we tested four genes: MACE-P004 (homolog of transmembrane protein gene in *M. truncatula, P. sativum* LGIII: 24.5); MACE-P009 (homolog of cathepsin B-like cysteine protease in *M. truncatula, P. sativum* LGV: 20.3); MACE-P015 (homolog of peptidoglycan-binding domain protein gene in *M. truncatula, P. sativum* LGIII: 103.2); and Shatterproof (homolog of MADS-box transcription factor in *M. truncatula, P. sativum* LGIII: 89.3). As a result we didn't find any trend in down or over expression in contrasting RILs in relationship to dehiscence levels, with only one exception of MACE-P015 (homolog of peptidoglycan-binding domain protein gene in *M. truncatula, P. sativum* LGIII: 103.2) which showed trend of down expression in all tested indehiscent RILs (Figure 10B) with only one exception—RIL 61 (JI 64 × JI 92). Base on screening of PCR length polymorphism we recognized this line 61 (F_6_) as heterozygous in MACE-P015 candidate gene because of presence of both parental alleles.

#### GO annotation, KEGG

To annotate the DEGs in dehiscence-parents and RILs, the consensus MACE sequences were searched against the NCBI non-redundant protein database by blastx using *e*-value cut off of 1e^−05^. In biological process, most DEGs were found to be involved in metabolic processes (37 genes, 34%) which includes cellular metabolic process (28 genes, 26%) and primary metabolic process (28 genes, 26%), pigmentation (8 genes, 7%), regulation of biological process (8 genes, 7%), developmental processes such as anatomical structure development (3 genes, 3%; Table S6). In molecular function, the maximum DEGs were found to be involved in catalytic activity which includes mainly hydrolase activity (12 genes, 11%), transferase activity (8 genes, 7%) and oxidoreductase activity (7 genes, 6%). Next, the DEGs were mostly found to be involved in binding related activities such as nucleic acid binding (10 genes, 9%) followed by nucleotide binding (9 genes, 8%) and nucleoside binding (5 genes, 5%). In the cellular component, the bulk of the DEGs belonged to cell part (41 genes, 38%) followed by membrane related proteins (22 genes, 20%), intracellular organelle (23 genes, 21%), and membrane-bound organelle (19 genes, 18%; Table S6). The KEGG pathway analysis showed that PsCam046431 is involved in phenylpropanoid biosynthesis, PsCam021037 in pentose phosphate, PsCam038465 in glycerophospholipid metabolism and PsCam042882 in carbon fixation pathways.

#### Genetic mapping of dehiscence locus

We assume that dehiscent and indehiscent RILs bulks are contrasting for genomic regions which are responsible for dehiscence trait, because of selection for this trait and repeated selfing of RILs lines. On the other hand remaining genomic regions should be represented by polymorphic reads from RILs libraries due to mixing of RILs lines during bulking. When we compared polymorphism in reads from indehiscent RILs bulk and dehiscent RILs bulk three homozygous SNP rich genomic region associated with dehiscent trait were identified due to identification of homozygous SNP which indicated that this region was under selection during RILs lines development (Figure S6). Based on sequence homology first of them is located in the second half of *M. truncatula* chromosome 1. Second and third region are more clearer in contrast with first region. Second region is located at the beginning of chromosome 2 where homozygous SNP are concentrated around 2 megabase and third region is situated around 53 megabase at the end of chromosome 3. Others homozygous SNP are spread across all *Medicago* chromosomes and do not form distinct cluster.

## Discussion

Plant domestication process is interesting phenomenon of accelerated human directed evolution. To dissect genetic changes associated with this process, either wild to cultivated crosses and linkage mapping or newly genome wide association mapping are employed to infer on number of genes governing domestication traits. Although some of the genes underlying domestication traits were shown to be regulated at transcriptional level (Doebley et al., 1997; Konishi et al., 2006) limited studies were conducted to investigate transcriptomic changes between wild progenitors and cultivated crops, analyzing pod or seed tissues, such as wheat glumes (Zou et al., 2015). We used comprehensive transcriptomic, metabolomics, and anatomical analyses to compare domesticated and wild pea seed coats and pods in relation to the loss of seed dormancy and pod dehiscence.

### Seed coat anatomical structure and histochemical properties

Histological analysis of the seed coat in *M. truncatula* revealed changes in cell wall thickness in the outer integuments throughout seed development (Verdier et al., 2013a). In *Arabidopsis* and *Melilotus* (legume), seed permeability was modulated by mutations affecting extracellular lipid biosynthesis (in Verdier et al., 2013a). Similarly, in *M. truncatula*, cells of the outer integument showed abundant accumulation of polyphenolic compounds (Figure 4); which upon oxidation may impact seed permeability (Moïse et al., 2005). Current knowledge about physical dormancy mainly comes from studies on morphological structure, phenolic content, and cuticle composition in legume species (reviewed in Smýkal et al., 2014). Morphological observation indicated that seed hardness was associated with the structure of palisade and cuticular layer (Vu et al., 2014) and presence or absence of cracks (Meyer et al., 2007; Koizumi et al., 2008). Other authors have proposed that the compositions of carbohydrates, hydroxylated fatty acids, or phenol compounds in seed coats control the level of permeability (Mullin and Xu, 2000, 2001; Shao et al., 2007; Zhou et al., 2010). Mullin and Xu (2001) found that the seed coat of an impermeable genotype had a high concentration of hemicellulose, essentially composed of xylans, which would reduce the hydrophilicity of the seed coat. We have found considerable structural and functional differences in testa properties between wild and domesticated peas. Contrary to Ma et al. (2004), who found small cuticular cracks in soft but not hard seeds of soybean, the surface was similar among used lines with only small discontinuities over the whole surface (Figure 3). However, we cannot exclude that these small fissures resulted from the SEM sample preparation, similarly to the above mentioned work. In the non-dormant genotypes subjected to imbibition, large fissures appear preferentially in the hilar region and strophiole (not shown). However, those are the most likely consequence of embryo imbibition and its volume increase and thus we do not expect those as primary sites of water entrance. There is a lack of detailed description of the primary pathway of water entry in pea as well as in the whole *Fabaceae* family, although the topic is thoroughly discussed (e.g., Baskin et al., 2000; Meyer et al., 2007; Ranathunge et al., 2010; Smýkal et al., 2014). It is thus not clear whether the hilar or strophiolar region is the primary entrance of water in the non-dormant genotypes as suggested also by McDonald et al. (1988) or Korban et al. (1981) in soybean and common bean (Agbo et al., 1987) or if the minor fissures present in the cuticle and properties of the outer part of palisade macrosclereids make the difference as suggested by Ma et al. (2004). Interesting and generally neglected feature of macrosclereids is a presence of autofluorescent, phenolics containing lipidic material in the terminal caps of macrosclereids above the light line, which is not directly connected with the cuticle (Figure S2). There is no detailed information on the nature of this material or its possible functional significance. Obviously, detailed structure of outer part of the palisade macrosclereids deserves future attention. Dormant genotypes have thicker macrosclereids palisade layer, which might contribute to the water impermeability of coats of dormant pea genotypes as suggested by Miao et al. (2001). However, thickness alone does not necessarily account for water impermeability (de Souza and Marcos-Filho, 2001). Our results also suggest different thickness of palisade layer among the dormant genotypes with the most dormant JI64 having the thickest palisade layer (Figure 2). Metachromatic staining with toluidine blue revealed that the non-dormant non-pigmented genotype Cameor exhibits high level of polyanionic pectins with exposed free carboxyl groups (Figure S3). On the other hand, the non-dormant, but well-pigmented JI92 showed lower abundance of pectins related anions, similarly to pigmented dormant genotypes. There is clear connection between toluidine blue stainability and seed coat pigmentation—anthocyanidin presence. Taken together results from toluidine and Alciane blue with vaniline and DMACA suggest that tannins in the seed coats of pigmented peas are probably bound to other compounds of the cell walls, changing the staining properties of cell walls. Nature of these linkages is unknown, but covalently bound tannins might be indicated as alkaline hydrolysis releases proanthocyanidins from the cell walls (Krygier et al., 1982). Such expectation might be further supported by presence quercetin-3-rhamnoside fragments from LC/MS and MALDI-MS study. The detected increase in metachromatic staining of testa cell walls after weak acid treatment might be indicative of a crosslinking of pectins with other compounds in dormant as well as JI92 genotype, which is released in acid environment. Interestingly, it was reported that condensed tannins might be released from linkages in acid environment (Porter, 1989). We can speculate whether the intensity of pectin—tannin crosslinking is associated with physical dormancy of pea. There are scarce references indicating possible role of tannins in cell wall polymer network and its properties (Pizzi and Cameron, 1986). There is some supposition for callose deposition in the light line area (e.g., de Souza et al., 2012). However, strong staining with aniline blue fluorochrome staining in the upper part of macrosclereids including the light line (Figure S2) cannot be attributed to callose. Such assumption is consistent with the specific callose antibody localization which led us to the conclusion that the signal for aniline blue is probably signal for one or more other compounds structurally similar to callose. The interaction of aniline blue fluorochrome with other 1,3- or 1,4-β-D-glucans was described by Evans et al. (1984) and this interaction depends on the degree of polymerization, and nature of substitution of the 1,3-β-D-glucan chain as well as on the concentration of phosphate in the staining solution (Evans et al., 1984). Deeper anatomical and histochemical analysis of seed coat and more reliable detection of primary entrance point of water during early rehydration phase is needed.

### Differentially expressed genes during seed coat development and seed dormancy

Seed development has been thoroughly studied in number of crops including legumes, especially with focus on embryo development (Bewley et al., 2013). In our study, we have made comparative transcriptomics analysis in order to dissect candidate genes/pathways associated with domestication imposed changes on seed coat properties. Temporal transcriptional changes during seed and pod development were studied in pigeonpea (Pazhamala et al., 2016), soybean (Aghamirzaie et al., 2015; Redekar et al., 2015), *Medicago* (Gallardo et al., 2007; Benedito et al., 2008; Verdier et al., 2013a; Righetti et al., 2015), peanut (Zhu et al., 2014; Wan et al., 2016), and pea (Liu et al., 2015) seeds but no study was made on comparison of wild progenitor and cultivated crop. Moreover, these studies analyzed either entire seed/pod or developing embryos, while in our work we have used excised seed coat or dissected pod suture. During the RNA isolation from the seed coat tissue, we have experienced great difficulties when working with wild pea seed samples. As reported earlier for pigmented soybean seeds (Wang and Vodkin, 1994) proanthocyanidins binds to RNA and prevent its extraction. We failed when using standard phenol/chloroform (McCarty, 1986) or guanidium thiocyanate (Chomczynski and Sacchi, 1987) methods, as well as common plant tissue RNA isolation kits.

There is problem of ambiguously mapped reads, which are the major source of error in RNA-Seq quantification (Robert and Watson, 2015). Short-read alignment is a complex problem due to the common occurrence of gene families. In contrast to RNA-seq, MACE methodology is derived from 3′ UTR end of transcript and each is represented by single molecule. The choice of quantification tool also has large effect, as these also differ in the way they handle aligned data and multi-mapped/ambiguous reads (Robert and Watson, 2015). In order to exclude or minimalize that identify DEGs are solely due to the genetic differences between contrasting parental genetic stocks we have used phenotypically classified bulks derived from RILs (Table S1, Figure S1). The concept of Bulk segregant analysis (BSA) was established as a method to detect markers in a specific genomic region by comparing two pooled DNA samples of individuals from a segregating population (Michelmore et al., 1991). Coupling BSA with the high throughput RNA sequencing has been shown to be an efficient tool for gene mapping and identification of differentially expressed genes (Chayut et al., 2015; Bojahr et al., 2016). One possible bottleneck of our analysis was bulking of several developmental stages into single MACE sample, where temporal and spatial expression might be hidden, resulting in differences between MACE and qRT-PCR data. Dynamic nature of gene expression both in spatial and temporal levels is clearly seen at qRT-PCR analysis of selected DEGs (Figures 9, 10). The bulking of various developmental stages, moreover from different genotypes (in case of RILs) is the source of imprecision which can result in masking of DEGs. The key to the successful use of BSA is precision of phenotypic assignment. Although some imprecision in phenotypic classification and comparable low number of RILs used for bulking (Table S1), transcriptomics analysis has provided valid results. As shown on heat-map (Figure 8), RIL bulks are indeed genetic mixture of parental genotypes. MACE method (Kahl et al., 2012) detects allele-specific SNPs and indels associated with the defined genotypes that can be instantly used in genetic mapping (Bojahr et al., 2016). As result, there was significant clustering of homozygous SNPs associated with seed dormancy (e.g., respective parental alleles) on *Mt* chromosomes 3 and 4, or chickpea chromosomes 5 and 7, respectively (not shown). These correspond to pea linkage groups (LG) III and IV. Using identical RIL mapping population and DARTseq markers, we mapped seed coat thickness to LG I, III, IV, and VI (unpublished) and percentage of seed germination to LGII. These indicate that there is likely more than single major gene involved in seed dormancy, acting at different stages (testa thickness, permeability).

Despite that detected DEGs between dormant and non-dormant pea seeds belongs to various GO and KEGG pathways, the largest number of annotated ones was found within phenylpropanoid and flavonoid pathways (Figure S4). These are involved in various activities such as UV filtration, fixing atmospheric nitrogen, and protection of cell walls (Zhao et al., 2013). Analysis of soybean mutant defective in seed coat led to identification of differentially expressed proline-rich and other cell wall protein transcripts (Kour et al., 2014). Moreover, this single gene mutation has resulted in differential expression of 1,300 genes, pointing out that complex series of events, many manifested at the transcript level, lead to changes in physiology, and ultimately structure of the cell wall. Similarly, we speculate that gene/-s causative of pea seed dormancy results in complex transcriptional and metabolomics changes. Recently, KNOTTED-like homeobox (KNOXII) gene, KNOX4, was found responsible for the loss of physical dormancy in the mutant *Medicago* seeds (Chai et al., 2016) resulting in differences in lipid monomer composition. These findings are in agreement with our data obtained by laser desorption-ionization mass spectrometric comparative analysis between dormant and non-dormant pea genotypes. Especially long chain hydroxylated fatty acids such as mono and dihydroxylated hexacosanoate, heptacosanoate, and octacosanoate were found in higher concentration in dormant peas compared to non-dormant ones (as shown in Figure 8 for dihydroxyheptacosanoate and dihydroxyoctacosanoate), implying that the presence of a greater proportion of hydroxylated fatty acids may provide a greater interconnectivity of cutin hydrophobic components improving its stability and impermeability for water as discussed also by Shao et al. (2007). As downstream targets of KNOX4 gene, several key genes related to cuticle biosynthesis were identified, such as the cytochrome P450-dependent fatty acid omega-hydroxylase and fatty acid elongase 3-ketoacyl-CoA synthase (Chai et al., 2016). We have not found homologs genes when searched within our DEGs set. It can be hypothesized that different genes have been altered in independently domesticated crops, although possibly acting on identical pathways. There are limited studies combining of transcriptomic and metabolomics analysis (Enfissi et al., 2010) or a combination of both techniques including proteomics (Barros et al., 2010; Collakova et al., 2013). Such integrative approach enables not just to identify transcript and metabolite changes associated with given process, but also to focus on biochemical pathways relevant to studied trait and possibly also delimit candidate genes. As shown in *Medicago* (Verdier et al., 2013a) and soybean (Ranathunge et al., 2010) the gene expression in seed coat is complex and dynamic. Since we could not currently annotate 10% of detected transcripts, it can be expected that with available pea genome this could be further improved. They might include putative target candidate/-s for seed coat permeability.

### Dormant pea seed coat accumulates more proanthocyanidins

In legume seeds, there are three parts: the seed coat, the cotyledon, and the embryonic axis which, on average, represent 10, 89, and 1%, respectively, of the seed content. Seed coat pigmentation was shown to correlate with imbibition ability in several legumes, including common bean (Caldas and Blair, 2009), chickpea (Legesse and Powell, 1996), yardlong bean (Kongjaimun et al., 2012), faba bean (Ramsay, 1997), and pea (Marbach and Mayer, 1974; Werker et al., 1979). The presence of proanthocynidins (PAs) in seed coats can be assessed by the appearance of brownish coloration, which is the result of PA oxidation by polyphenol oxidase (Marles et al., 2008). In soybean (*Glycine max*), the recessive *i* allele results in high anthocyanin accumulation in the seed coat, resulting in dark brown or even black color (Tuteja et al., 2004; Yang et al., 2010). In contrast, the dominant *I* allele, which silences chalcone synthase (CHS) expression and hence blocks both anthocyanin and PA biosynthesis, results in a completely colorless seed coat. However, there is not simple relationship between the testa pigmentation imposing dormancy, as numerous cultivated pea varieties have colored testa yet do not display seed dormancy as illustrated by this study used JI92 landrace. Mendel's *A* gene beside flower color has pleiotropic effect including seed coat pigmentation (Hellens et al., 2010), yet these traits can be decoupled by recombination (Smýkal, unpublished). Second, *B* gene of pea encodes a defective flavonoid 3′, 5′-hydroxylase, and confers pink flower color, by control of hydroxylation of flavonoid precursors (Moreau et al., 2012). Neither this mutation results in alteration of seed dormancy. Comparably more is known on *Arabidopsis* and *Medicago* seed development owing to available mutants. Many of these mutations indicate the important role of proanthocyanidins and flavonoid pigments in testa development (Graeber et al., 2012) including effect on seed dormancy. The proanthocyanidins (PAs) received particular attention due to their abundance in seed coats (Dixon et al., 2005; Zhao et al., 2010) including pea (Ferraro et al., 2014). PAs are also known as the chemical basis for tannins, which are considered to be important part of physical dormancy in some species (Kantar et al., 1996; Ramsay, 1997). Flavan-3-ol-derived PA oligomers and anthocyanins are derived from the same precursors, proanthocyanidins (Lepiniec et al., 2006) and chemical diversity is introduced early in the pathway by cytochrome P450 enzymes (reviewed in Li et al., 2016a). Anthocyanidin synthase, anthocyanidin reductase, and leucoanthocyanidin reductase were studied at transcriptional level by Ferraro et al. (2014) in cultivated pea varieties and showed to be developmentally regulated. In our comparative transcriptome profiling we have not found any of these genes to be among DEGs, suggesting that differences in metabolites (quercetin, gallocatechin) found by chemical analysis are not at these steps of PA biosynthesis. Anthocyanidins are either immediately modified by glycosylation to give anthocyanins by anthocyanidin 3-O-glycosyltransferases (UGTs) or reduced to generate flavan-3-ols (such as epicatechin) by anthocyanidin reductase for PA biosynthesis (Xie et al., 2003, 2004). There are several described *Medicago* mutants defective in respective genes, resulting in reduced testa pigmentation (Li et al., 2016b), although the relationship to seed dormancy was not specifically investigated. Indeed, we have detected several differentially expressed UDP-glycosylases, two of them studied by qRT-PCR (Figure 9). The glycosyltransferase superfamily consists of 98 subfamilies and only few have been characterized so far. Only a few members of the UGT72 family been shown to have activity toward flavonoids; such as the seed coat-specific UGT72L1 from *Medicago* (Zhao et al., 2010) and several seed specific UGTs in *L. japonicus* (Yin et al., 2017). The UGT72L1 catalyzes (Zhao et al., 2010) formation of epicatechin 3′-O-glucoside (E3′OG), the preferred substrate for MATE transporters. MATE1 mutant display altered seed coat structure and PAs accumulation (Zhao and Dixon, 2011) and also has significantly lower seed dormancy levels (Smýkal, unpublished). The mechanism of PA polymerization is still unclear, but may involve the laccase-like polyphenol oxidase (Zhao et al., 2010). Notably, *Medicago myb5* and *myb14* mutants exhibit darker seed coat color than wild-type plants, with *myb5* also showing deficiency in mucilage biosynthesis, and accumulating only of the PA content of wild-type plants. When *myb5* seeds are exposed to water, they germinate readily without dormancy typical of wild type *Medicago* seeds (R. Dixon, personal communication and P. Smýkal, unpublished). All these observations suggest that PA oligomers play indeed a role in seed coat mediated dormancy.

Our LC/ESI-MS experiments confirm the presence of significantly higher contents of dimer and trimer of gallocatechin (i.e., soluble tannins of prodelphinidin type) in dormant compared to non-dormant pea genotypes. Catechin dimer and trimer was also found in the pea seed coat extracts but their differences between dormant and non-dormant peas are much less significant than their gallocatechin counterparts. This fact points out the significance of hydroxylation of B-ring of PAs in relation to dormancy. The insoluble PAs are the result of oxidative cross-linking with other cell components. Variation in PA content in the pea seeds has been reported (Troszyńska and Ciska, 2002) but not in comparison of wild vs. cultivated peas. PAs play also important roles in defense to pathogens, and because of the health benefits are of industry and medicine interest. PA biosynthesis and its regulation have been dissected in *Arabidopsis* using *transparent testa* (*tt*) mutants, which regulate production, transport or storage of PAs (Lepiniec et al., 2006), and 20 genes affecting flavonoid metabolism were characterized at the molecular level (reviewed in Bradford and Nonogaki, 2009). Many of these flavonoid biosynthesis pathway genes have been found to affect dormancy of *Arabidopsis* seeds, indicating the role of pigments in this process (Debeaujon et al., 2000). Similarly *Medicago* also synthesizes PAs in the seed coat, which consists essentially of epicatechin units (Lepiniec et al., 2006; Zhao et al., 2010). Polymerization of soluble phenolics to insoluble polymers is promoted by peroxidases (Gillikin and Graham, 1991) and catecholoxidases (Marbach and Mayer, 1974; Werker et al., 1979), which are abundant in legume seed coats. Positive correlation in content of phenolics, the requirement of oxidation and the activity of catechol oxidase in relation to seed dormancy (germination) in wild vs. domesticated pea seeds have been shown by Marbach and Mayer (1974) and Werker et al. (1979). Recently, epicatechin, cyanidin 3-O-glucoside, and delphinidin 3-O-glucoside were isolated in wild compared to cultivated soybean seed coats (Zhou et al., 2010) with epicatechin being in significant positive correlation with hardseededness.

Beside proanthocyanidin also flavonols, first of all quercetin derivatives, are frequently found in legumes including pea (Dueñas et al., 2004). We found significantly higher content of quercetin rhamnoside in dormant JI64 genotype compared to non-dormant JI92. Similarly, its hydroxylated analog, i.e., myricetin-3-rhamnoside appeared to be a marker of dormancy. This compound was found in many legumes including chickpea, horse gram (Sreerama et al., 2010) and pea (Dueñas et al., 2004). As described in review of Agati et al. (2012) the antioxidant properties of flavonoids represent a robust biochemical trait of organisms exposed to oxidative stress of different origin during plant-environment interactions (regulation of the action of reaction oxygen species, ROS). The effect of ROS on the plant developmental processes including seed germination was described by Singh et al. (2016) including increase of free radical scavenging during pea seed germination (Lopez-Amoros et al., 2006). Presence of phenolic compounds in seed coat might help to protect against fungal diseases during germination as shown in lentil (Matus and Slinkard, 1993).

### Pod dehiscence

Pod maturation might terminate with pod shattering, which is an important trait for seed dispersal of wild species but generally unwanted trait in crops (Fuller and Allaby, 2009). Central to the ballistic seed dispersal in *Pisum* is the dehiscent pod (single carpel fused along its edges) where the central pod suture undergoes an explosive rupturing along a dehiscence zone (Ambrose and Ellis, 2008). During pod shattering, the two halves of the pod detach due to a combination of the diminished cell walls adhesion in the dehiscence zone, and the tensions established by the specific mechanical properties of drying cells of endo and exocarp of the pod shell. These two principal aspects are shared among families producing dry dehiscent fruit, such as *Fabaceae* and *Brassicaceae* (Grant, 1996; Dong and Wang, 2015). The spring-like tension within the pod shell is generated during differential drying-induced shrinkage of endocarp and the outer part of the shell—exocarp (Armon et al., 2011). Properties of both the obliquely arranged rigid and lignified inner sclerenchyma as well as the pectin rich exocarp cell with longitudinal orientation are crucial factors of springing in Fabaceae pods. Regulators of their development affecting geometrical arrangement of the layers and their histological properties (cell wall thickness and composition, lignification, hydration) will be key factors generating required tension. Composition and characteristics of pod cell shell cell walls correlate with shattering of yardlong bean and wild cowpea (Suanum et al., 2016) and thickness of the shell and extend of sclerenchymatous dorsal bundle caps was connected with shattering in soy (Tiwari and Bhatia, 1995). The major QTL controlling pod dehiscence in soybean is *qPDH1* (QTL for Pod Dehiscence 1). *qPDH1* had been recently cloned and shown to encode a dirigent-like protein expressed in the sclerenchyma of differentiating endocarp and modulating the mechanical properties of the pod shell. Lignin biosynthesis is the most likely process affected by *qPHD1* (Suzuki et al., 2010; Funatsuki et al., 2014), which might be connected with modulation of torsion within drying pod walls (Funatsuki et al., 2014). However, the precise biochemical activity of *qPHD1* is still unclear. Similarly the sclerenchyma differentiation and lignification of the endocarp and valve margin cells of Arabidopsis are central to silique dehiscence with *NAC SECONDARY WALL THICKENING PROMOTING FACTOR 1 (NST1)* and *SECONDARY WALL ASSOCIATED NAC DOMAIN PROTEIN 1 (SDN1)* being identified as master regulators of their differentiation (Zhong et al., 2010). Lignification of endocarp and valve margin cells is lost together with dehiscence in the *nst1snd1* double mutant (Mitsuda and Ohme-Takagi, 2008). The other decisive point of pod dehiscence is mechanical stability/instability of dehiscence (suture) zone which might trigger the explosive release of pod shell tension (Grant, 1996). Decreased cell to cell adhesion, might be carried out by action of endo-1,4-glucanases and endopolygalacturonases disintegrating the middle lamella in the separation layer (Christiansen et al., 2002). Degradation of pectin in the middle lamella of abscission zone is common theme in fruit shattering (Dong and Wang, 2015). Contrary, mechanism of pod shattering resistance due to reinforcement of the suture has been described in domesticated soybeans *NST1/2* homologous transcription factor *SHATTERING1-5* (*SHAT1-5*) from NAC family has been unveiled, inducing excessive secondary cell wall deposition and lignification in the outer part of the suture (fiber cap cells; Dong et al., 2014). Up-regulated expression of *SHAT1-5* in domesticated soy “locks” the dehiscence zone interconnecting the vascular bundle caps of sclerenchymatous fibers in the suture vicinity preventing shattering.

The fundamental elements of fruit shattering regulatory network is being uncovered recently in *Arabidopsis* and homologous genes were identified also in other species and crops (*Zea mays, Triticum aestivum, Oryza sativa, Glycine max, Sorghum bicolor, Sorghum propinquum*, or *Solanum lycopersicum*; for review see Dong and Wang, 2015; Ballester and Ferrandiz, 2016). MADS box genes of *Arabidopsis SHATTERPROOF1 (SHP1)* and *SHATTERPROOF2 (SHP2)* participate in the dehiscence zone specification (Liljegren et al., 2000). *INDEHISCENT (IND)* b-HLH transcription factor act down-stream to SHP1/2 as regulator sclerenchyma differentiation in endocarp and valve margin. The *shp1/2* double mutant as well as *ind* produces indehiscent siliques devoid of proper cell specification and differentiation in the dehiscence zone (Liljegren et al., 2004; Dong and Wang, 2015). SHP1 and SHP2 are required for the proper specification of the different cell types within the valve margin and the DZ and both genes probably represent the top of the hierarchy regulating DZ formation (Liljegren et al., 2000). *SHATTERPROOF* genes along with *INDEHISCENT* (*IDEH*) are the main regulators of establishment of lignified layer, which causes pod dehiscence. Another MADS box gene involved in dehiscence zone formation is *FRUITFULL* (*FUL*), which expression appears at the inception of the carpel primordia, and soon after becomes restricted to the cells that will give rise to the valves, a pattern that is complementary to that of the SHP genes (Liljegren et al., 2004; Dong and Wang, 2015). Significant up-regulation of *SHATTERING1–5* (*SHAT1–5*) in the fiber cap cells (FCC) of cultivated soybean was shown to be responsible for the excessive cell wall deposition in the FCC, which in turn prevents the pod from committing dehiscence after maturation (Dong et al., 2014). Homologous genes defining dehiscence zone identity and its differentiation *INDEHISCENT, SPATULA, SHATTERPROOF, bZIP*, and *SHATTERING* (Ferrándiz et al., 2000; Girin et al., 2011; Dong et al., 2014) were identified in *Pisum* genome and their abundance was tested in RNA isolated from pod suture tissue of wild and domesticated pea as well as of contrast RILs incurred from crossing of wild and domesticate parent with dehiscent or indehiscent pods. In case of *SHATTERPROOF* and *SHATTERING* homologous genes, we found differences in expression between parental lines of pea but we didn't find the similar results in case of contrast RILs. The other homologous genes (*INDEHISCENT, SPATULA*, and *bZIP*) did now exhibit any significant difference in expression between dehiscent and non-dehiscent phenotypes.

In the legumes, the pod shattering trait is controlled by one or two dominant genes or QTL. In pea and lentil, genes controlling pod shattering map to a syntenic region, suggesting that the same genes may have been modified during the domestication of the two cool-season legumes (Weeden et al., 2002; Weeden, 2007). Single locus control of pod dehiscence was found in lentil (Ladizinsky, 1998), while two loci in mungbean (Isemura et al., 2012), yardlong bean (Kongjaimun et al., 2012), one controlling the number of twists along the length of the shattered pod, and second the percentage of shattered pods, similarly to two loci found in pea (Weeden et al., 2002; Weeden, 2007), and common bean (Koinange et al., 1996). Bordat et al. (2011) localized *Dpo* locus responsible for loss of pea pod dehiscence on LGIII. We obtained the similar result by the genome-wide DArTseq analysis. Based on comparison of our candidate gene position in *M. truncatula* genome and SNPs map (Tayeh et al., 2015) we localized our candidate genes in pea genome. In total of our 25 candidate genes 7 are localized on LGIII, 6 on LGVI, 4 on LGII, 3 on LGV, 2 on LGVII, 2 on LGIV, and 1 on LGI (not shown). MACE-P015, the main candidate gene possibly responsible for pod dehiscence localized on LGIII, is a homolog of peptidoglycan-binding domain protein (PGDB) of *M. truncatula* (Medtr2g079050). These proteins may have a general peptidoglycan binding function and this motif is found at the N or C terminus of a variety of enzymes involved in bacterial cell wall degradation. Many of the proteins having this domain are so far uncharacterized. Matrix metalloproteinases (MMP), which catalyze extracellular matrix degradation, have N-terminal domains that resemble PGBD (Seiki, 1999). On the other hand our candidate MACE-P015 has also 80% match with *Cicer arietinum* proline-rich extensin-like protein EPR1 (XM_004488673). Extensins are plant specific structural cell-wall proteins (Lamport et al., 2011); they can account for up to 20% of the dry weight of the cell wall and can significantly modulate mechanical cell wall properties through linkages to other cell wall component, which can play a role in pod dehiscence.

### New insights into pea seed and pod development in relation to domestication

Study of biochemical and molecular mechanisms underlying plant domestication process is important area of research in plant biology. In the current study we used a comparative anatomy, metabolomics, and transcriptome profiling of pods and seed coats in wild and domesticated pea in order to identify genes associated with loss of seed dormancy as well as pod dehiscence. We have identified genes showing differential expression in respective parents as well as phenotypically contrasting RILs. Among others, there were number of genes belonging to phenylpropanoid pathway, which was also identified by metabolomics analysis of seed coat. Our results support the role of proanthocyanidins and their derivatives in physical seed coat mediated dormancy. One of the identified differentially expressed gene involved in pod dehiscence showed significant down-expression in dorsal and ventral pod suture of indehiscent genotypes. Moreover, this homolog of peptidoglycan-binding domain or proline-rich extensin-like protein mapped correctly to predicted *Dpo1* locus on PsLGIII. This integrated analysis of the seed coat in wild and cultivated pea raised new questions associated with domestication and seed dormancy. Having underlying gene(s) in hands for various independently domesticated legume crops it would help our understanding of genetic and molecular processes involved in seeds dormancy. Moreover, extended knowledge on control seed dispersal and seed dormancy is necessary for diverse applications—biodiversity conservation as well as breeding.

## Author contributions

Conceived and designed experiments: PS, PB, AS, and PH. Performed experiments: IH, PS, AJ, PH, LP, MC, and MV. Analyzed the data: IH, AJ, PB, OT, and KA. Wrote the paper: IH, AJ, OT, PH, and PS. All authors have read and approved the manuscript.

### Conflict of interest statement

The authors declare that the research was conducted in the absence of any commercial or financial relationships that could be construed as a potential conflict of interest.

## Supplementary material

Table S1 **Germination characteristics of parental and RIL lines**.

Table S2 **Summary statistics for MACE sequencing data and annotation to the Pisum mRNA sequences from NCBI and to the contigs based on the *de novo* assembly of sequences**.

Table S3 **List of primers used for qRT-PCR**.

Table S4 **Complete set of DEGs detected in seed dormancy and pod dehiscence experiments, between wild and cultivated parental genotypes as well as RILs, with indicated homology to annotated pea transcriptome (Tayeh et al., 2015) and MACE expression values**.

Table S5 **GO annotations of seed dormancy genes**.

Table S6 **GO annotations of pod dehiscence genes**.

Figure S1 **Germination indexes of all 126 RIL lines tested dat 25C over the period of 7 days**. Ordered by cummulative germination percentages **(A)** with shown Coefficient of velocity **(B)**, Timson indexes **(C)**, and Mean germination time **(D)** arranged accordingly.

Figure S2 **Seed coat transverse sections from extrahilar region:** Cameor **(a)**, JI92 **(b)**, JI64 **(c)**, and VIR320 **(d)**. UV excited autofluorescence (**a–d):** white arrow, cuticle; asterisk, light line. Sudan Red 7B staining of terminal parts of macrosclereids **(e–h)**: white arrow, cuticle; black arrow, lipidic material different from the cuticle). Aniline blue fluorochrome staining under UV excitation **(i–l)**: asterisk, light line; white arrow, the edge between pigmented and non-pigmented interface of JI92, inlay in **(i)** callose immunodetection (blue excitation); scale bars = 25 μm)

Figure S3 **Seed coat transverse sections from extrahilar region:** Cameor **(a)**, JI92 **(b)**, JI64 **(c)**, and VIR320 **(d)**. Metachromatic toluidine blue staining is indicative of high density of polyanionic surface **(a–d)**: asterisk, light line. Toluidine blue staining after mild acid treatment **(e–h):** black arrow = the edge between pigmented and non-pigmented interface of JI92; scale bar = 50 μm.

Figure S4 **KEGG phenylpropanoid (A)** and flavonoid **(B)** pathways of DEGs between dormant and nondormant seeds.

Figure S5 **KEGG phenylpropanoid pathway of DEGs between dehiscent and indehiscent pods**.

Figure S6 **Strictly homozygous SNP between dehiscence and indehiscence RIL bulks mapped to the eight *Medicago truncatula* chromosomes**.

## References

[B1] Abbo S. Rachamim E. Zehavi Y. Zezak I. Lev-Yadun S. Gopher A. (2011). Experimental growing of wild pea in Israel and its bearing on near Eastern plant domestication. Ann. Bot. 107, 1399–1404. 10.1093/aob/mcr081 21527420 PMC3101147

[B2] Agati G. Azzarello E. Pollastri S. Tattini M. (2012). Flavonoids as antioxidants in plants: location and functional significance. Plant Sci. 196, 67–76. 10.1016/j.plantsci.2012.07.014 23017900

[B3] Agbo G. N. Hosfield M. A. Uebersax M. A. Klomparens K. (1987). Seed microstructure and its relationship to water uptake in isogenic lines and a cultivar of drybeans (*Phaseolus vulgaris* L.). Food Microstruct. 6, 91–102.

[B4] Aghamirzaie D. Batra D. Heath L. S. Schneider A. Grene R. Collakova E. (2015). Transcriptome-wide functional characterization reveals novel relationships among differentially expressed transcripts in developing soybean embryos. BMC Genomics 16:928. 10.1186/s12864-015-2108-x 26572793 PMC4647491

[B5] Alves-Carvalho S. Aubert G. Carrere S. Cruaud C. Brochot A.-L. Jacquin F. (2015). Full-lenght *de novo* assembly of RNAseq data in pea (*Pisum sativum* L.) provides a gene expression atlas and gives insights in root nodulation in this species. Plant J. 8, 1–19. 10.1111/tpj.12967 26296678

[B6] Amarowicz R. Estrella I. Hernández T. Dueñas M. Troszyńska A. Kosińska A.. (2009). Antioxidant activity of a red lentil extract and its fractions. Int. J. Mol. Sci. 10, 5513–5527. 10.3390/ijms10125513 20054484 PMC2802008

[B7] Ambrose M. J. Ellis T. H. N. (2008). Ballistic seed dispersal and associated seed shadow in wild Pisum germplasm. Pisum Genet. 40, 5–10.

[B8] Appelhagen I. Thiedig K. Nordholt N. Schmidt N. Huep G. Sagasser M.. (2014). Update on transparent testa mutants from *Arabidopsis thaliana*: characterisation of new alleles from an isogenic collection. Planta 240, 955–970. 10.1007/s00425-014-2088-0 24903359

[B9] Armon S. Efrati E. Kupferman R. Sharon E. (2011). Geometry and mechanics in the opening of chiral seed pods. Science 333, 1726–1730. 10.1126/science.1203874 21940888

[B10] Bajaj D. Das S. Upadhyaya H. D. Ranjan R. Badoni S. Kumar V.. (2015). A genome-wide combinatorial strategy dissects complex genetic architecture of seed coat color in chickpea. Front. Plant Sci. 6:979. 10.3389/fpls.2015.00979 26635822 PMC4647070

[B11] Ballester P. Ferrandiz C. (2016). Shattering fruits: variations on a dehiscent theme. Curr. Opin. Plant Biol. 35, 68–75. 10.1016/j.pbi.2016.11.008 27888713

[B12] Barros E. Lezar S. Anttonen M. J. van Dijk J. P. Röhlig R. M. Kok E. J.. (2010). Comparison of two GM maize varieties with a near-isogenic non-GM variety using transcriptomics, proteomics and metabolomics. Plant Biotechnol. J. 8, 436–451. 10.1111/j.1467-7652.2009.00487.x 20132517

[B13] Baskin J. M. Baskin C. C. (2004). A classification system for seed dormancy. Seed Sci. Res. 14, 1–16. 10.1079/SSR2003150

[B14] Baskin J. M. Baskin C. C. Li X. (2000). Taxonomy, anatomy and evolution of physical dormancy in seeds. Plant Species Biol. 15, 139–152. 10.1046/j.1442-1984.2000.00034.x

[B15] Beisson F. Li Y. Bonaventure G. Pollard M. Ohlrogge J. B. (2007). The acyltransferase GPAT5 is required for the synthesis of suberin in seed coat and root of *Arabidopsis*. Plant Cell 19, 351–368. 10.1105/tpc.106.048033 17259262 PMC1820950

[B16] Benedito V. A. Torres-Jerez I. Murray J. D. Andriankaja A. Allen S. Kakar K.. (2008). A gene expression atlas of the model legume *Medicago truncatula*. Plant J. 55, 504–513. 10.1111/j.1365-313X.2008.03519.x 18410479

[B17] Bennett E. J. Roberts J. A. Wagstaff C. (2011). The role of the pod in seed development: strategies for manipulating yield. New Phytol. 190, 838–853. 10.1111/j.1469-8137.2011.03714.x 21507003

[B18] Bewley J. D. (1997). Seed germination and dormancy. Plant Cell 9, 1055–1066. 10.1105/tpc.9.7.1055 12237375 PMC156979

[B19] Bewley J. D. Bradford K. Hilhorst H. Nonogaki H. (2013). Seeds: Physiology of Development, Germination and Dormancy, 3rd Edn. NewYork, NY: Springer-Verlag 10.1007/978-1-4614-4693-4

[B20] Bogdanova V. S. Galieva E. R. Yadrikhinskiy A. K. Kosterin O. E. (2012). Inheritance and genetic mapping of two nuclear genes involved in nuclear–cytoplasmic incompatibility in peas (*Pisum sativum* L.). Theor. Appl. Genet. 124, 1503–1512. 10.1007/s00122-012-1804-z 22318398

[B21] Bojahr J. Nhengiwa O. Krezdorn N. Rotter B. Saal B. Ruge-Wehling B.. (2016). Massive analysis of cDNA ends (MACE) reveals a co-segregating candidate gene for *LpPg1* stem rust resistance in perennial ryegrass (*Lolium perenne*). Theor. Appl. Genet. 129, 1915–1932. 10.1007/s00122-016-2749-4 27435735

[B22] Bordat A. Savois V. Nicolas M. Salse J. Chauveau A. Bourgeois M.. (2011). Translational genomics in legumes allowed placing *in silico* 5460 unigenes on the pea functional map and identified candidate genes in *Pisum sativum* L. G3 1, 93–103. 10.1534/g3.111.000349 22384322 PMC3276132

[B23] Bradford K. Nonogaki H. (2009). Seed Development, Dormancy and Germination. Annual Plant Review, Vol. 27 Oxford: Blackwell.

[B24] Caldas G. V. Blair M. W. (2009). Inheritance of condensed tannin content and relationship with seed colour and pattern genes in common bean (*Phaseolus vulgaris* L.). Theor. Appl. Genet. 119, 131–142. 10.1007/s00122-009-1023-4 19363663

[B25] Chai M. Zhou C. Molina I. Fu C. Nakashima J. Li G.. (2016). A class II KNOX gene, KNOX4, controls seed physical dormancy. Proc. Natl. Acad. Sci. U.S.A. 113, 6997–7002. 10.1073/pnas.1601256113 27274062 PMC4922145

[B26] Chayut N. Yuan H. Ohali S. Meir A. Yeselson Y. Portnoy V.. (2015). A bulk segregant transcriptome analysis reveals metabolic and cellular processes associated with Orange allelic variation and fruit β-carotene accumulation in melon fruit. BMC Plant Biol. 15:274. 10.1186/s12870-015-0661-8 26553015 PMC4640158

[B27] Chen H. Osuna D. Colville L. Lorenzo O. Graeber K. Küster H.. (2013). Transcriptome-wide mapping of pea seed ageing reveals a pivotal role for genes related to oxidative stress and programmed cell death. PLoS ONE 8:e78471. 10.1371/journal.pone.0078471 24205239 PMC3812160

[B28] Chomczynski P. Sacchi N. (1987). Single-step method of RNA isolation by acid guanidinium thiocyanate-phenol-chloroform extraction. Anal. Biochem. 162, 156–159. 2440339 10.1006/abio.1987.9999

[B29] Christiansen L. C. Dal Degan F. Ulvskov P. Borkhardt B. (2002). Examination of the dehiscence zone in soybean pods and isolation of a dehiscence-related endopolygalacturonase gene. Plant Cell Environ. 25, 479–490. 10.1046/j.1365-3040.2002.00839.x

[B30] Collakova E. Aghamirzaie D. Fang Y. Klumas C. Tabataba F. Kakumanu A.. (2013). Metabolic and transcriptional reprogramming in developing soybean (*Glycine max*) embryos. Metabolites 3, 347–372. 10.3390/metabo3020347 24957996 PMC3901275

[B31] de Souza F. H. D. Marcos-Filho J. (2001). A seed coat as a modulator of seed-environment relationships in Fabaceae. Rev. Bras. Bot. 24, 365–375. 10.1590/s0100-84042001000400002

[B32] de Souza T. V. Voltolini C. H. Santos M. Silveira Paulilo M. T. (2012). Water absorption and dormancy-breaking requirements of physically dormant seeds of *Schizolobium parahyba* (Fabaceae–Caesalpinioideae). Seed Sci. Res. 22, 169–176. 10.1017/S0960258512000013

[B33] Debeaujon I. Léon-Kloosterziel K. M. Koornneef M. (2000). Influence of the testa on seed dormancy, germination, and longevity in *Arabidopsis*. Plant Physiol. 122, 403–414. 10.1104/pp.122.2.403 10677433 PMC58877

[B34] Dixon R. A. Sharma S. B. Xie D. (2005). Proanthocyanidins—a final frontier in flavonoid research? New Phytol. 165, 9–28. 10.1111/j.1469-8137.2004.01217.x 15720617

[B35] Doebley J. Stec A. Hubbard L. (1997). The evolution of apical dominance in maize. Nature 386, 485–488. 10.1038/386485a0 9087405

[B36] Dong Y. Wang Y.-Z. (2015). Seed shattering: from models to crops. Front. Plant Sci. 6:476. 10.3389/fpls.2015.00476 26157453 PMC4478375

[B37] Dong Y. Yang X. Liu J. Wang B. H. Liu B. L. Wang Y. Z.. (2014). Pod shattering resistance associated with domestication is mediated by a NAC gene in soybean. Nat. Commun. 5:3352. 10.1038/ncomms4352 24549030

[B38] Duarte J. Riviere N. Baranger A. Aubert G. Burstin J. Cornet L.. (2014). Transcriptome sequencing for high throughput SNP development and genetic mapping in pea. BMC Genomics 15:126. 10.1186/1471-2164-15-126 24521263 PMC3925251

[B39] Dueñas M. Estrella I. Hernández T. (2004). Occurrence of phenolic compounds in the seed coat and the cotyledon of peas (*Pisum sativum* L.). Eur. Food Res. Technol. 219, 116–123. 10.1007/s00217-004-0938-x

[B40] Enfissi E. M. Barneche F. Ahmed I. Lichtlé C. Gerrish C. McQuinm R. P.. (2010). Integrative transcript and metabolite analysis of nutritionally enhanced DE-ETIOLATED1 downregulated tomato fruit. Plant Cell 22, 1190–1215. 10.1105/tpc.110.073866 20435899 PMC2879742

[B41] Evans N. A. Hoyne P. A. Stone B. A. (1984). Characteristics and specificity of the interaction of a fluorochrome from aniline blue (sirofluor) with polysaccharides. Carbohydr. Polym. 4, 215–230. 10.1016/0144-8617(84)90012-2

[B42] Ferrándiz C. Liljegren S. J. Yanofsky M. F. (2000). Negative regulation of the SHATTERPROOF genes by FRUITFULL during Arabidopsis fruit development. Science 289, 436–438. 10.1126/science.289.5478.436 10903201

[B43] Ferraro K. Jin A. L. Nguyen T.-R. Reinecke D. M. Ozga J. A. Ro D.-K. (2014). Characterization of proanthocyanidin metabolism in pea (*Pisum sativum*) seeds. BMC Plant Biol. 14:238. 10.1186/s12870-014-0238-y 25928382 PMC4175280

[B44] Finch-Savage W. E. Leubner-Metzger G. (2006). Seed dormancy and the control of germination. New Phytol. 171, 501–523. 10.1111/j.1469-8137.2006.01787.x 16866955

[B45] Foyer C. H. Lam H. M. Nguyen H. T. Siddique K. H. M. Varshney R. K. Colmer T. D.. (2016). Neglecting legumes has compromised human health and sustainable food production. Nature Plants 2, 1–10. 10.1038/nplants.2016.112 28221372

[B46] Franssen S. U. Shrestha R. P. Bräutigam A. Bornberg-Bauer E. Weber A. P. (2011). Comprehensive transcriptome analysis of the highly complex *Pisum sativum* genome using next generation sequencing. BMC Genomics 12:227. 10.1186/1471-2164-12-227 21569327 PMC3224338

[B47] Fuller D. Q. Allaby R. G. (2009). Seed dispersal and crop domestication: shattering, germination and seasonality in evolution under cultivation. Ann. Plant Rev. 38, 238–295. 10.1002/9781444314557.ch7

[B48] Fuller D. Q. Denham T. Arroyo-Kalin M. Lucas M. Stevens C. J. Qin L.. (2014). Convergent evolution and parallelism in plant domestication revealed by an expanding archaeological record. Proc. Natl. Acad. Sci. U.S.A. 111, 6147–6152. 10.1073/pnas.1308937110 24753577 PMC4035951

[B49] Funatsuki H. Suzuki M. Hirose A. Inaba H. Yamada T. Hajika M.. (2014). Molecular basis of a shattering resistance boosting global dissemination of soybean. Proc. Natl. Acad. Sci. U.S.A. 111, 17797–17802. 10.1073/pnas.1417282111 25468966 PMC4273335

[B50] Gallardo K. Firnhaber C. Zuber H. Héricher D. Belghazi M. Henry C.. (2007). A combined proteome and transcriptome analysis of developing *Medicago truncatula* seeds. Mol. Cell. Proteomics 6, 2165–2179. 10.1074/mcp.M700171-MCP200 17848586

[B51] Gardner R. O. (1975). Vanillin - hydrochloric acid as a histochemical test for tannin. Stain Technol. 50, 315–317. 10.3109/10520297509117081 54955

[B52] Gillikin J. W. Graham J. S. (1991). Purification and developmental analysis of the major anionic peroxidases from the seed coat of *Glycine max*. Plant Physiol. 96, 214–220. 10.1104/pp.96.1.214 16668154 PMC1080735

[B53] Girin T. Paicu T. Stephenson P. Fuentes S. Korner E. O'Brien M.. (2011). INDEHISCENT and SPATULA interactto specify carpel and valve margin tissue and thus promote seed dispersal in *Arabidopsis*. Plant Cell 23, 3641–3653. 10.1105/tpc.111.090944 21990939 PMC3229140

[B54] Graeber K. Nakabayashi K. Miatton E. Leubner-Metzger G. Soppe W. J. J. (2012). Molecular mechanisms of seed dormancy. Plant Cell Environ. 35, 1769–1786. 10.1111/j.1365-3040.2012.02542.x 22620982

[B55] Grant W. F. (1996). Seed pod shattering in the genus *Lotus* (Fabaceae): a synthesis of diverse evidence. Can. J. Plant Sci. 76, 447–456. 10.4141/cjps96-079

[B56] Hammer K. (1984). Das Domestikationssyndrom. Kulturpflanze 32, 11–34. 10.1007/BF02098682

[B57] Hedley C. L. Smith C. M. Ambrose M. J. Cook S. Wang T. L. (1986). An analysis of seed development in *Pisum sativum*. The effect of the r-locus on the growth and development of the seed. Ann. Bot. 58, 371–379. 10.1093/oxfordjournals.aob.a087215

[B58] Hellens R. P. Moreau C. Lin-Wang K. Schwinn K. E. Thomson S. J. Fiers M. W.. (2010). Identification of Mendel's white flower character. PLoS ONE 5:e13230. 10.1371/journal.pone.0013230 20949001 PMC2952588

[B59] Isemura T. Kaga A. Tabata S. Somta P. Srinives P. Shimizu T.. (2012). Construction of a genetic linkage map and genetic analysis of domestication related traits in mungbean (*Vigna radiata*). PLoS ONE 7:e41304. 10.1371/journal.pone.0139666 22876284 PMC3410902

[B60] Jang S. J. Sato M. Sato K. Jitsuyama Y. Fujino K. Mori H.. (2015). A single-nucleotide polymorphism in an endo-1,4-β-glucanase gene controls seed coat permeability in soybean. PLoS ONE 10:e0128527. 10.1371/journal.pone.0128527 26039079 PMC4454576

[B61] Kahl G. Molina C. Rotter B. Jüngling R. Frank A. Krezdorn N. (2012). Reduced representation sequencing of plant stress transcriptomes. J. Plant Biochem. Biotechnol. 21, 119–127. 10.1007/s13562-012-0129-y

[B62] Kalendar R. Lee D. Schulman A. H. (2014). FastPCR software for PCR, *in silico* PCR, and oligonucleotide assembly and analysis, in DNA Cloning and Assembly Methods, Vol. 1116, Methods in Molecular Biology, eds Valla S. Lale R. (New York, NY: Springer), 271–302. 10.1007/978-1-62703-764-8_18 24395370

[B63] Kantar F. Pilbeam C. J. Hebblethwaite P. D. (1996). Effect of tannin content of faba bean (*Vicia faba*) seed on seed vigour, germination and field emergence. Ann. Appl. Biol. 128, 85–93. 10.1111/j.1744-7348.1996.tb07092.x

[B64] Kaur S. Pembleton L. W. Cogan N. O. Savin K. W. Leonforte T. Paull J.. (2012). Transcriptome sequencing of field pea and faba bean for discovery and validation of SSR genetic markers. BMC Genomics 13:104. 10.1186/1471-2164-13-104 22433453 PMC3352077

[B65] Kislev M. E. Bar-Yosef O. (1988). The legumes: the earliest domesticated plants in the Near East? Curr. Anthropol. 29, 175–179. 10.1086/203623

[B66] Koinange E. M. K. Singh S. Gepts P. (1996). Genetic control of the domestication syndrome in common bean. Crop Sci. 36, 1037–1045. 10.2135/cropsci1996.0011183X003600040037x

[B67] Koizumi M. Kaori K. Seiichiro I. Nobuaki I. Shighiro N. Hiromi K. (2008). Role of seed coat in imbibing soybean seed observed by micro-magnetic resonance imaging. Ann. Bot. 102, 343–352. 10.1093/aob/mcn095 18565982 PMC2516911

[B68] Kongjaimun A. Kaga A. Tomooka N. Somta P. Vaughan D. A. Srinives P. (2012). The genetics of domestication of yardlongbean, *Vigna unguiculata* (L.) Walp. ssp. *unguiculata* cv.-gr. *sesquipedalis*. Ann. Bot. 109, 1185–1200. 10.1093/aob/mcs048 22419763 PMC3336956

[B69] Konishi S. Izawa T. Lin S. Y. Ebana K. Fukuta Y. Sasaki T.. (2006). An SNP caused loss of seed shattering during rice domestication. Science 312, 1392–1396. 10.1126/science.1126410 16614172

[B70] Korban S. S. Coyne D. P. Weihing J. L. (1981). Evaluation, variation, and genetic control of seed coat whiteness in dry beans (*Phaseolus vulgaris* L.). J. Am. Soc. Hortic. Sci. 166, 575–579.

[B71] Kour A. Boone A. M. Vodkin L. O. (2014). RNA-Seq profiling of a defective seed coat mutation in *Glycine max* reveals differential expression of proline-rich and other cell wall protein transcripts. PLoS ONE 9:e96342. 10.1371/journal.pone.0096342 24828743 PMC4020777

[B72] Krygier K. Frank Sosulski F. Hogge L. (1982). Free, esterified, and insoluble-bound phenolic acids. 1. Extraction and purification procedure. J. Agric. Food Chem. 30, 330–334. 10.1021/jf00110a028

[B73] Kučera L. Kurka O. Barták P. Bednář P. (2017). Liquid chromatography/high resolution tandem mass spectrometry - tool for the study of polyphenol profile changes during micro-scale biogas digestion of grape marcs. Chemosphere 166, 463–472. 10.1016/j.chemosphere.2016.09.124 27710883

[B74] Kujur A. Bajaj D. Upadhyaya H. D. Das S. Ranjan R. Shree T.. (2015). A genome-wide SNP scan accelerates trait-regulatory genomic loci identification in chickpea. Sci. Rep. 5:11166. 10.1038/srep11166 26058368 PMC4461920

[B75] Ladizinsky G. (1998). Plant Evolution under Domestication. Dordrecht: Kluwer Academic Publishers.

[B76] Lamport D. T. A. Kieliszewski M. J. Chen Y. Cannon M. C. (2011). Role of the Extensin superfamily in primary cell wall architecture. Plant Physiol. 156, 11–19. 10.1104/pp.110.169011 21415277 PMC3091064

[B77] Legesse N. Powell A. A. (1996). Relationship between the development of seed coat pigmentation, seed coat adherence to the cotyledons and the rate of imbibition during the maturation of grain legumes. Seed Sci. Technol. 24, 23–32.

[B78] Lenser T. Theißen G. (2013). Molecular mechanisms involved in convergent crop domestication. Trends Plant Sci. 18, 704–714. 10.1016/j.tplants.2013.08.007 24035234

[B79] Lepiniec L. Debeaujon I. Routaboul J. M. Baudry A. Pourcel L. Nesi N.. (2006). Genetics and biochemistry of seed flavonoids. Annu. Rev. Plant Biol. 57, 405–430. 10.1146/annurev.arplant.57.032905.105252 16669768

[B80] Li P. Chen B. Zhang G. Chen L. Dong Q. Wen J.. (2016b). Regulation of anthocyanin and proanthocyanidin biosynthesis by *Medicago truncatula* bHLH transcription factor MtTT8. New Phytol. 210, 905–921. 10.1111/nph.13816 26725247

[B81] Li P. Dong Q. Ge S. He X. Verdier J. Li D.. (2016a). Metabolic engineering of proanthocyanidin production by repressing the isoflavone pathways and redirecting anthocyanidin precursor flux in legume. Plant Biotechnol. J. 14, 1604–1618. 10.1111/pbi.12524 26806316 PMC5066740

[B82] Li Y.-G. Tanner G. Larkin P. (1996). The DMACA–HCl protocol and the threshold proanthocyanidin content for bloat safety in forage legumes. J. Science Food Agric. 70, 89–101. 10.1002/(SICI)1097-0010(199601)70:1<89::AID-JSFA470>3.0.CO;2-N

[B83] Liljegren S. J. Ditta G. S. Eshed Y. Savidge B. Bowman J. Yanofsky M. F. (2000). SHATTERPROOF MADS-box genes control seed dispersal in *Arabidopsis*. Nature 404, 766–770. 10.1038/35008089 10783890

[B84] Liljegren S. J. Roeder A. H. Kempin S. A. Gremski K. Østergaard L. Guimil S.. (2004). Control of fruit patterning in *Arabidopsis* by INDEHISCENT. Cell 116, 843–853. 10.1016/S0092-8674(04)00217-X 15035986

[B85] Liu C. Jun J. H. Dixon R. A. (2014). MYB5 and MYB14 play pivotal roles in seed coat polymer biosynthesis in *Medicago truncatula*. Plant Physiol. 165, 1424–1439. 10.1104/pp.114.241877 24948832 PMC4119029

[B86] Liu N. Zhang G. Xu S. Mao W. Hu Q. Gong Y. (2015). Comparative transcriptomic analyses of vegetable and grain pea (*Pisum sativum* L.) seed development. Front. Plant Sci. 6:1039. 10.3389/fpls.2015.01039 26635856 PMC4658420

[B87] Lopez-Amoros M. L. Hernandez T. Estrella I. (2006). Effect of germination on legume phenolic compounds and their antioxidant activity. J. Food Compost. Anal. 19, 277–283. 10.1016/j.jfca.2004.06.012

[B88] Ma F. Cholewa E. Mohamed T. Peterson C. A. Gijzen M. (2004). Cracks in the palisade cuticle of soybean seed coats correlate with their permeability to water. Ann. Bot. 94, 213–228. 10.1093/aob/mch133 15217785 PMC4242158

[B89] Marbach I. Mayer A. M. (1974). Permeability of seed coats to water as related to drying conditions and metabolism of phenolics. Plant Physiol. 54, 817–820. 10.1104/pp.54.6.817 16658981 PMC366614

[B90] Marles M. A. S. Vandenberg A. Bett K. E. (2008). Polyphenol oxidase activity and differential accumulation of polyphenolics in seed coats of pinto bean (*Phaseolus vulgaris* L.) characterize postharvest color changes. J. Agric. Food Chem. 56, 7049–7056. 10.1021/jf8004367 18666779

[B91] Matus A. Slinkard A. E. (1993). Effect of fungicidal seed treatment on zero-tannin lentil. Lens Newsl. 20, 46–50.

[B92] McCarty D. R. (1986). A simple method for extraction of DNA from maize tissue. Maize Genet. Coop. Newsl. 60, 61.

[B93] McDonald M. B. Vertucci C. W. Roos E. E. (1988). Soybean seed imbibition: water absorption by seed parts. Crop Sci. 28, 993–997. 10.2135/cropsci1988.0011183X002800060026x

[B94] Meyer C. J. Steudle E. Peterson C. A. (2007). Patterns and kinetics of water uptake by soybean seeds. J. Exp. Bot. 58, 717–732. 10.1093/jxb/erl244 17185739

[B95] Meyer R. S. Purugganan M. D. (2013). Evolution of crop species: genetics of domestication and diversification. Nat. Rev. Genet. 14, 840–852. 10.1038/nrg3605 24240513

[B96] Meyer R. S. DuVal A. E. Jensen H. R. (2012). Patterns and processes in crop domestication: an historical review and quantitative analysis of 203 global food crops. New Phytol. 196, 29–48. 10.1111/j.1469-8137.2012.04253.x 22889076

[B97] Miao Z. H. Fortune J. A. Gallagher J. (2001). Anatomical structure and nutritive value of lupin seed coats. Aust. J. Agric. Res. 52, 985–993. 10.1071/AR00117

[B98] Michelmore R. W. Paran I. Kesseli R. V. (1991). Identification of markers linked to disease-resistance genes by bulked segregant analysis: a rapid method to detect markers in specific genomic regions by using segregating populations. Proc. Natl. Acad. Sci. U.S.A. 88, 9828–9832. 10.1073/pnas.88.21.9828 1682921 PMC52814

[B99] Mitsuda N. Ohme-Takagi M. (2008). NAC transcription factors NST1 and NST3 regulate pod shattering in a partially redundant manner by promoting secondary wall formation after the establishment of tissue identity. Plant J. 56, 768–778. 10.1111/j.1365-313X.2008.03633.x 18657234

[B100] Moïse J. A. Han S. Gudynaite-Savitch L. Johnson D. A. Miki B. L. A. (2005). Seed coats: structure, development, composition, and biotechnology. In vitro Cell. Dev. Biol. Plant 41, 620–644. 10.1079/IVP2005686

[B101] Moreau C. Mike J. Ambrose M. J. Turner L. Hill L. Ellis T. H. N.. (2012). The b gene of pea encodes a defective flavonoid 3′,5′-hydroxylase, and confers pink flower color. Plant Physiol. 159, 759–768. 10.1104/pp.112.197517 22492867 PMC3375939

[B102] Mullin W. J. Xu W. (2000). A study of the intervarietal differences of cotyledon and seed coat carbohydrates in soybean. Food Res. Int. 33, 883–891. 10.1016/S0963-9969(00)00118-6

[B103] Mullin W. J. Xu W. (2001). Study of soybean seed coat components and their relationship to water absorption. J. Agric. Food Chem. 49, 5331–5335. 10.1021/jf010303s 11714324

[B104] Nikolaeva M. G. (1969). Physiology of Deep Dormancy in Seeds. Leningrad, Russia, Izdatelstvo ‘Nauka’. Transl. by Shapiroin Russian Z. Washington, DC: National Science Foundation.

[B105] Nonogaki H. (2014). Seed dormancy and germination – emerging mechanisms and new hypotheses. Front. Plant Sci. 5:233. 10.3389/fpls.2014.00233 24904627 PMC4036127

[B106] North N. Casey R. Domoney C. (1989). Inheritance and mapping of seed lipoxygenase polypeptides in Pisum. Theor. Appl. Genet. 77, 805–808. 10.1007/BF00268330 24232895

[B107] Olsen K. M. Wendel J. F. (2013). A bountiful harvest: genomic insights into crop domestication phenotypes. Annu. Rev. Plant Biol. 64, 47–70. 10.1146/annurev-arplant-050312-120048 23451788

[B108] Patil G. Valliyodan B. Deshmukh R. Prince S. Nicander B. Zhao M. (2015). Soybean (*Glycin max*) SWEET gene family: insight through comparative genomics, transcriptome profiling and whole genome re-sequence analysis. BMC Genomics 16:520 10.1186/s12864-015-1730-y 26162601 PMC4499210

[B109] Pazhamala L. T. Agarwal G. Bajaj P. Kumar V. Kulshreshtha A. Saxena R. K.. (2016). Deciphering transcriptional programming during pod and seed development using RNA-Seq in pigeonpea (*Cajanus cajan*). PLoS ONE 11:e0164959. 10.1371/journal.pone.0164959 27760186 PMC5070767

[B110] Pizzi A. Cameron F. A. (1986). Flavonoid tannins — structural wood components for drought-resistance mechanisms of plants. Wood Sci. Technol. 20:119.

[B111] Porter L. J. (1989). Condensed tannins, in Natural Products of Woody Plants I: Chemicals Extraneous to the Lignocellulosic Cell, ed Rowe J. W. (Berlin: Springer), 651–690. 10.1007/978-3-642-74075-6_18

[B112] Pradhan S. Bandhiwal N. Shah N. Kant C. Gaur R. Bhatia S. (2014). Global transctriptome analysis of developing chickpea (*Cicer arietinum* L.) seeds. Front. Plant Sci. 5:698. 10.3389/fpls.2014.00698 25566273 PMC4267183

[B113] Purugganan M. D. Fuller D. Q. (2009). The nature of selection during plant domestication. Nature 457, 843–848. 10.1038/nature07895 19212403

[B114] Radchuk V. Borisjuk L. (2014). Physical, metabolic and developmental functions of the seed coat. Front. Plant Sci. 5:510. 10.3389/fpls.2014.00510 25346737 PMC4193196

[B115] Ramsay G. (1997). Inheritance and linkage of a gene for testa imposed seed dormancy in faba bean (*Vicia faba* L.). Plant Breed. 116, 287–289. 10.1111/j.1439-0523.1997.tb00998.x

[B116] Ranal M. A. Santana D. G. (2006). How and why to measure the germination process? Rev. Bras. Bot. 29, 1–11. 10.1590/S0100-84042006000100002

[B117] Ranathunge K. Shao S. Qutob D. Gijzen M. Peterson C. A. Bernards M. A. (2010). Properties of the soybean seed coat cuticle change during development. Planta 231, 1171–1188. 10.1007/s00425-010-1118-9 20186427

[B118] Redekar N. R. Biyashev R. M. Jensen R. V. Helm R. F. Grabau E. A. Maroof M. A. (2015). Genome-wide transcriptome analyses of developing seeds from low and normal phytic acid soybean lines. BMC Genomics 16:1074. 10.1186/s12864-015-2283-9 26678836 PMC4683714

[B119] Righetti K. Vu J. L. Pelletier S. Vu B. L. Glaab E. Lalanne D.. (2015). Inference of longevity-related genes from a robust coexpression network of seed maturation identifies regulators linking seed storability to biotic defense-related pathways. Plant Cell 27, 2692–2708. 10.1105/tpc.15.00632 26410298 PMC4682330

[B120] Robert C. Watson M. (2015). Errors in RNA-Seq quantification affect genes of relevance to human disease. Genome Biol. 16:177. 10.1186/s13059-015-0734-x 26335491 PMC4558956

[B121] Roth A. Ding J. Morin R. Crisan A. Ha G. Giuliany R.. (2012). JointSNVMix: a probabilistic model for accurate detection of somatic mutations in normal/tumour paired next-generation sequencing data. Bioinformatics 28, 907–913. 10.1093/bioinformatics/bts053 22285562 PMC3315723

[B122] Schmutz J. McClean P. E. Mamidi S. Wu G. A. Cannon S. B. Grimwood J.. (2014). A reference genome for common bean and genome-wide analysis of dual domestications. Nat. Genet. 46, 707–713. 10.1038/ng.3008 24908249 PMC7048698

[B123] Seiki M. (1999). Membrane-type matrix metalloproteinases. Acta Pathol. Microbiol. Immunol. Scand. 107, 137–143. 10190290 10.1111/j.1699-0463.1999.tb01536.x

[B124] Severin A. Woody J. L. Bolon Y.-T. Joseph B. Diers B. W. Farmer A. D.. (2010). RNA-Seq atlas of *Glycine max*: a guide to the soybean transcriptome. BMC Plant Biol. 10:160. 10.1186/1471-2229-10-160 20687943 PMC3017786

[B125] Shao S. Meyer C. J. Ma F. Peterson C. A. Bernards M. A. (2007). The outermost cuticle of soybean seeds: chemical composition and function during imbibition. J. Exp. Bot. 58, 1071–1082. 10.1093/jxb/erl268 17218545

[B126] Shi J. Lai J. (2015). Patterns of genomic changes with crop domestication and breeding. Curr. Opin. Plant Biol. 24, 47–53. 10.1016/j.pbi.2015.01.008 25656221

[B127] Singh R. Singh S. Parihar P. Mishra R. K. Tripathi D. K. Singh V. P. (2016). Reactive oxygen species (ROS): beneficial companions of plant's developmental processes. Front. Plant Sci. 7:1299 10.3389/fpls.2016.01299 27729914 PMC5037240

[B128] Smartt J. (1990). Grain Legumes: Evolution and Genetic Resources. Cambridge, UK: Cambridge University Press 10.1017/cbo9780511525483

[B129] Smýkal P. Aubert G. Burstin J. Coyne C. J. Ellis N. T. H. Flavell A. J. (2012). Pea (*Pisum sativum* L.) in the genomic era. Agronomy 2, 74–115. 10.3390/agronomy2020074

[B130] Smýkal P. Coyne C. J. Ambrose M. J. Maxted N. Schaefer H. Blair M. W. (2015). Legume crops phylogeny and genetic diversity for science and breeding. Crit. Rev. Plant Sci. 34, 43–104. 10.1080/07352689.2014.897904

[B131] Smýkal P. Vernoud V. Blair M. W. Soukup A. Thompson R. D. (2014). The role of the testa during development and in establishment of dormancy of the legume seed. Front. Plant Sci. 5:351. 10.3389/fpls.2014.00351 25101104 PMC4102250

[B132] Soukup A. (2014). Selected simple methods of plant cell wall histochemistry and staining for light microscopy, in Plant Cell Morphogenesis: Methods and Protocols, Vol. 1080, Methods in Molecular Biology, eds Žársky V. Cvrčková F. (New York, NY: Springer), 25–40. 10.1007/978-1-62703-643-6_2 24132416

[B133] Soukup A. Tylová E. (2014). Essential methods of plant sample preparation for light microscopy, in Plant Cell Morphogenesis: Methods and Protocols, Vol. 1080, Methods in Molecular Biology, eds Žársky V. Cvrčková F. (New York, NY: Springer), 1–23. 10.1007/978-1-62703-643-6_1 24132415

[B134] Sreerama N. Y. Sashikala V. B. Pratape V. M. (2010). Variability in the distribution of phenolic compounds in milled fractions of chickpea and horse gram: evaluation of their antioxidant properties. J. Agric. Food Chem. 58, 8322–8330. 10.1021/jf101335r 20593828

[B135] Suanum W. Somta P. Kongjaimun A. Yimram T. Kage A. Tomooka N. (2016). Co-localization of QTLs for pod fiber content and pod shattering in F-2 and backcross populations between yardlong bean and wild cowpea. Mol. Breeding 36, 80 10.1007/s11032-016-0505-8

[B136] Sudheesh J. Sawbridge T. I. Cogan N. O. Kennedy P. Forster J. W. Kaur S. (2015). *De novo* assembly and characterization of field pea transcriptome using RNA-Seq. BMC Genomics 16:611 10.1186/s12864-015-1815-7 26275991 PMC4537571

[B137] Sun L. Miao Z. Cai C. Zhang D. Zhao M. Wu Y.. (2015). GmHs1-1, encoding a calcineurin-like protein, controls hard-seededness in soybean. Nat. Genet. 47, 939–943. 10.1038/ng.3339 26098868

[B138] Suzuki M. Fujino K. Nakamoto Y. Ishimoto M. Funatsuki H. (2010). Fine mapping and development of DNA markers for the *qPDH1* locus associated with pod dehiscence in soybean. Mol. Breed. 25, 407–418. 10.1007/s11032-009-9340-5

[B139] Tayeh N. Aluome C. Falque M. Jacquin F. Klein A. Chauveau A.. (2015). Development of two major resources for pea genomics: the GenoPea 13.2K SNP Array and a high density, high resolution consensus genetic map. Plant J. 84, 1257–1273. 10.1111/tpj.13070 26590015

[B140] Tiwari S. P. Bhatia V. S. (1995). Characters of pod anatomy associated with resistance to pod-shattering in soybean. Ann. Bot. 76, 483–485. 10.1006/anbo.1995.1123

[B141] Troszyńska A. Ciska E. (2002). Phenolic compounds of seed coats of white and coloured varieties of pea (*Pisum sativum* L.) and their total antioxidant activity. Czech J. Food Sci. 20, 15–22.

[B142] Trygg J. Wold S. (2002). Orthogonal projections to latent structures (O-PLS). J. Chemometrics 16, 119–128. 10.1002/cem.695

[B143] Tuteja J. H. Clough S. J. Chan W. C. Vodkin L. O. (2004). Tissue specific gene silencing mediated by anaturally occurring chalcone synthase gene cluster in *Glycine max*. Plant Cell 16, 819–835. 10.1105/tpc.021352 15064367 PMC412859

[B144] Verdier J. Dessaint F. Schneider C. Abirached-Darmency M. (2013a). A combined histology and transcriptome analysis unravels novel questions on *Medicago truncatula* seed coat. J. Exp. Bot. 64, 459–470. 10.1093/jxb/ers304 23125357 PMC3542040

[B145] Verdier J. Torres-Jerez I. Wang M. Andriankaja A. Allen S. N. He J.. (2013b). Establishment of the *Lotus japonicus* gene expression atlas (LjGEA) and its use to explore legume seed maturation. Plant J. 74, 351–362. 10.1111/tpj.12119 23452239

[B146] Vu D. T. Velusamy V. Park E. (2014). Structure and chemical composition of wild soybean seed coat related to its permeability. Pak. J. Bot. 46, 1847–1857.

[B147] Wan L. Li B. Pandey M. K. Wu Y. Lei Y. Yan L.. (2016). Transcriptome analysis of a new peanut seed coat mutant for the physiological regulatory mechanism involved in seed coat cracking and pigmentation. Front. Plant Sci. 7:1491. 10.3389/fpls.2016.01491 27790222 PMC5063860

[B148] Wang C. S. Vodkin L. O. (1994). Extraction of RNA from tissues containing high levels of proanthocyanidins that bind RNA. Plant Mol. Biol. Rep. 12, 132–145.

[B149] Wang H. L. Grusak M. A. (2005). Structure and development of *Medicago truncatula* pod wall and seed coat. Ann. Bot. 95, 737–747. 10.1093/aob/mci080 15703184 PMC4246729

[B150] Wang L. Feng Z. Wang X. Wang X. Zhang X. (2010). DEGseq: an R package for identifying differentially expressed genes from RNA-seq data. Bioinformatics 26, 136–138. 10.1093/bioinformatics/btp612 19855105

[B151] Weeden N. F. (2007). Genetic changes accompanynig the domestication of *Pisum sativum*: is there a common genetic basis to the Domestication syndrome for legumes? Ann. Bot. 100, 1017–1025. 10.1093/aob/mcm122 17660515 PMC2759201

[B152] Weeden N. F. Brauner S. Przyborowski J. A. (2002). Genetic analysis of pod dehistance in pea (*Pisum sativum* L.). Cell. Mol. Biol. Lett. 7, 657–663. 12378224

[B153] Weitbrecht K. Muller K. Leubner-Metzger G. (2011). First off the mark: early seed germination. J. Exp. Bot. 62, 3289–3309. 10.1093/jxb/err030 21430292

[B154] Werker E. Marbach I. Mayer A. M. (1979). Relation between the anatomy of the testa, water permeability and the presence of phenolics in the genus *Pisum*. Ann. Bot. 43, 765–771. 10.1093/oxfordjournals.aob.a085691

[B155] Xie D. Y. Sharma S. B. Dixon R. A. (2004). Anthocyanidin reductases from *Medicago truncatula* and *Arabidopsis thaliana*. Arch. Biochem. Biophys. 422, 91–102. 10.1016/j.abb.2003.12.011 14725861

[B156] Xie D. Y. Sharma S. B. Paiva N. L. Ferreira D. Dixon R. A. (2003). Role of anthocyanidin reductase, encoded by BANYULS in plant flavonoid biosynthesis. Science 299, 396–399. 10.1126/science.1078540 12532018

[B157] Yang K. Jeong N. Moon J. Lee Y. H. Lee S. H. Kim H. M.. (2010). Genetic analysis of genes controlling natural variation of seed coat and flower colors in soybean. J. Heredity 101, 757–768. 10.1093/jhered/esq078 20584753

[B158] Yin Q. Shen G. Chang Z. Tang Y. Gao H. Pang Y. (2017). Involvement of three putative glucosyltransferases from the UGT72 family in flavonol glucoside/rhamnoside biosynthesis in *Lotus japonicus* seeds. J. Exp. Bot. 68, 597–612. 10.1093/jxb/erw420 28204516 PMC5444469

[B159] Young M. D. Wakefield M. J. Smyth G. K. Oshlack A. (2010). Gene ontology analysis for RNA-Seq: accounting for selection bias. Genome Biol. 11:R14. 10.1186/gb-2010-11-2-r14 20132535 PMC2872874

[B160] Zawada A. M. Rogacev K. Müller S. Rotter B. Winter P. Fliser D.. (2014). Massive analysis of cDNA ends (MACE) and miRNA expression profiling identifies proatherogenic pathways in chronic kidney disease. Epigenetics 9, 161–172. 10.4161/epi.26931 24184689 PMC3928179

[B161] Zhang S. Shi Y. Cheng N. Du H. Fan W. Wang C. (2015). *De novo* characterization of fall dormant and nondormant alfalfa (*Medicago sativa* L.) leaf transcriptome and identification of candidate genes related to fall dormancy. PLoS ONE 10:e0122170. 10.1371/journal.pone.0122170 25799491 PMC4370819

[B162] Zhao J. Pang Y. Dixon R. A. (2010). The mysteries of proanthocyanidin transport and polymerization. Plant Physiol. 153, 437–443. 10.1104/pp.110.155432 20388668 PMC2879784

[B163] Zhao Q. Dixon R. A. (2011). Transcriptional networks for lignin biosynthesis: more complex than we thought? Trends Plant Sci. 16, 227–233. 10.1016/j.tplants.2010.12.005 21227733

[B164] Zhao S. Tuan P. A. Li X. Kim Y. B. Kim H. Park C. G.. (2013). Identification of phenylpropanoid biosynthetic genes and phenylpropanoid accumulation by transcriptome analysis of *Lycium chinense*. BMC Genomics 14:802. 10.1186/1471-2164-14-802 24252158 PMC4046672

[B165] Zhong R. Lee C. Ye Z. (2010). Evolutionary conservation of the transcriptional network regulating secondary cell wall biosynthesis. Trend Plant Sci. 15, 625–632. 10.1016/j.tplants.2010.08.007 20833576

[B167] Zhou S. Sekizuka H. Yang Z. Sawa S. Pan J. (2010). Phenolics in the seed coat of wild soybean (*Glycine soja*) and their significance for seed hardness and seed germination. J. Agric. Food Chem. 58, 10972–10978. 10.1021/jf102694k 20879792

[B168] Zhou Z. Jiang Y. Wang Z. Gou Z. Lyu J. Li W.. (2015). Resequencing 302 wild and cultivated accessions identifies genes related to domestication and improvement in soybean. Nature Biotech. 33, 408–414. 10.1038/nbt.3096 25643055

[B169] Zhu W. Chen X. Li H. Zhu F. Hong Y. Varshney R. K.. (2014). Comparative transcriptome analysis of aerial and subterranean pods development provides insights into seed abortion in peanut. Plant Mol. Biol. 85, 395–409. 10.1007/s11103-014-0193-x 24793121 PMC4152868

[B170] Zhukov V. A. Zhernakov A. I. Kulaeva O. A. Ershov N. I. Borisov A. Y. Tikhonovich I. A. (2015). *De novo* assembly of the Pea (*Pisum sativum* L.) nodule transcriptome. Int. J. Genomics 2015:695947. 10.1155/2015/695947 26688806 PMC4672141

[B171] Zohary D. Hopf M. (2000). Domestication of Plants in the Old World, 3rd Edn. New York, NY: Oxford University Press.

[B172] Zou H. Tzarfati R. Hübner S. Krugman T. Fahima T. Abbo S.. (2015). Transcriptome profiling of wheat glumes in wild emmer, hulled landraces and modern cultivars. BMC Genomics 16:777. 10.1186/s12864-015-1996-0 26462652 PMC4603339

