# Supplementary figures and images for: A Combined Comparative Transcriptomic, Metabolomic, and Anatomical Analyses of Two Key Domestication Traits: Pod Dehiscence and Seed Dormancy in Pea (Pisum sp.)
Source: Front Plant Sci. 2017 Apr 25;8:542. doi: 10.3389/fpls.2017.00542 (PMC5404241; doi:10.3389/fpls.2017.00542)

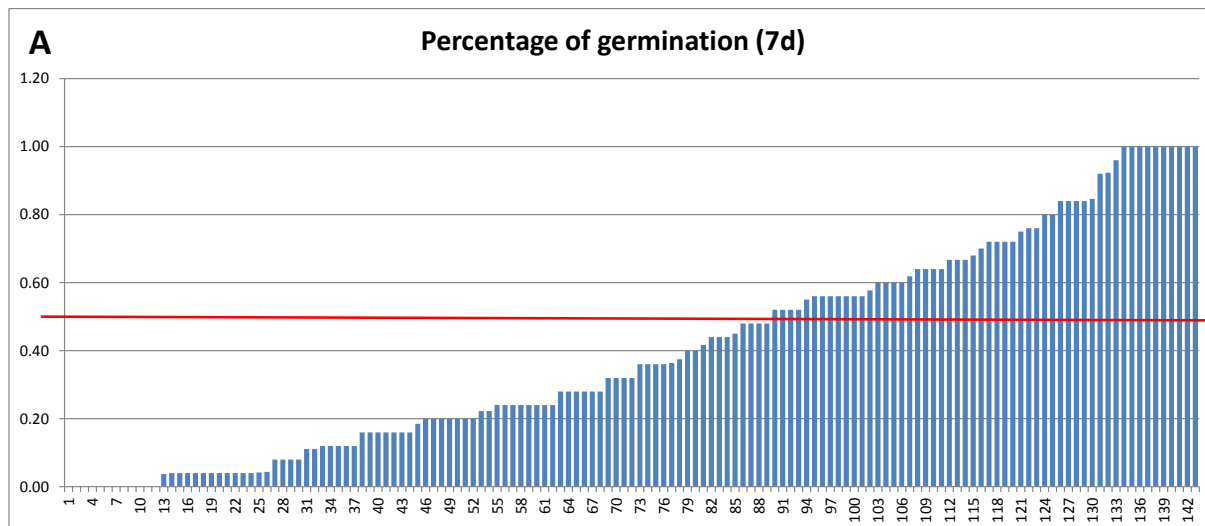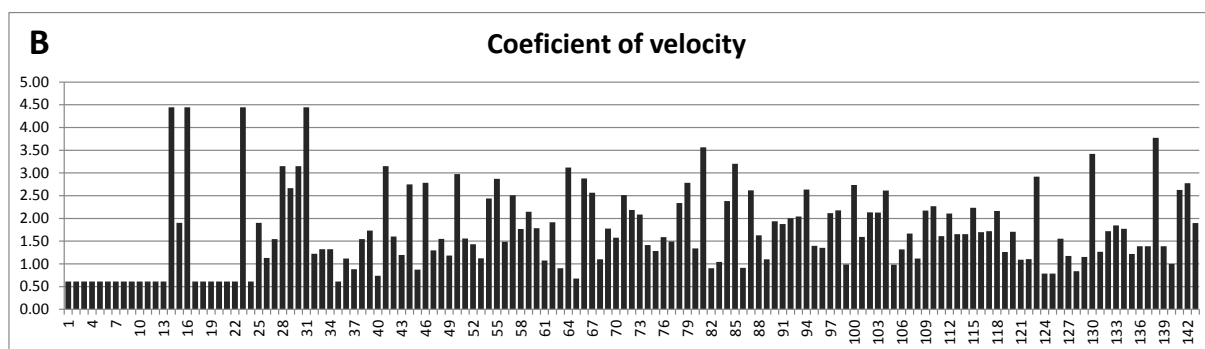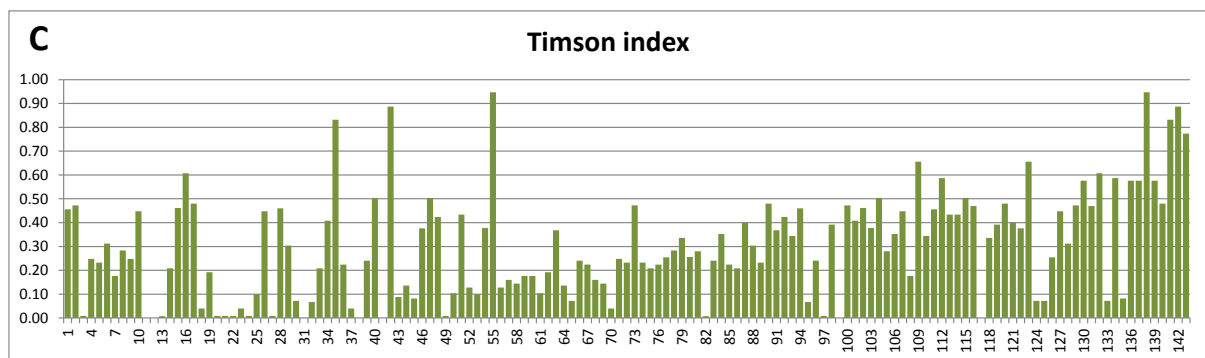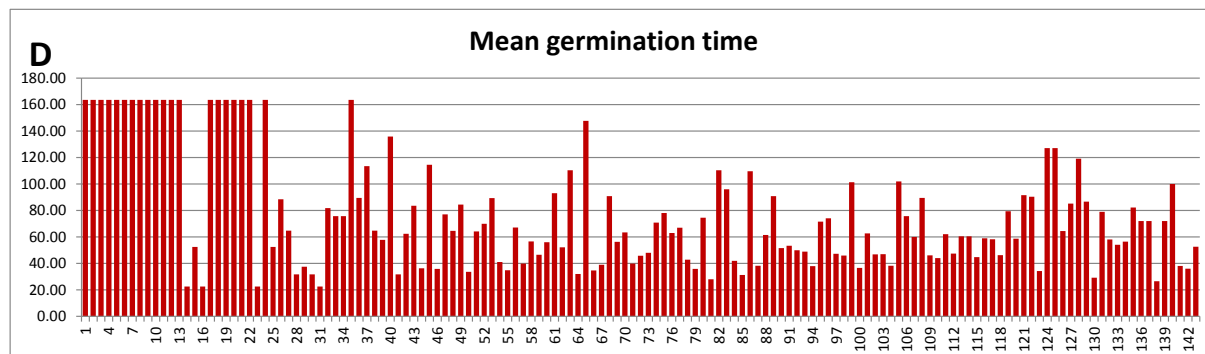

Supplement: Figure S1 — Germination indexes of all 126 RIL lines tested dat 25C over the period of 7 days. Ordered by cummulative germination percentages (A) with shown Coefficient of velocity (B), Timson indexes (C), and Mean germination time (D) arranged accordingly. [file Image1.PDF]

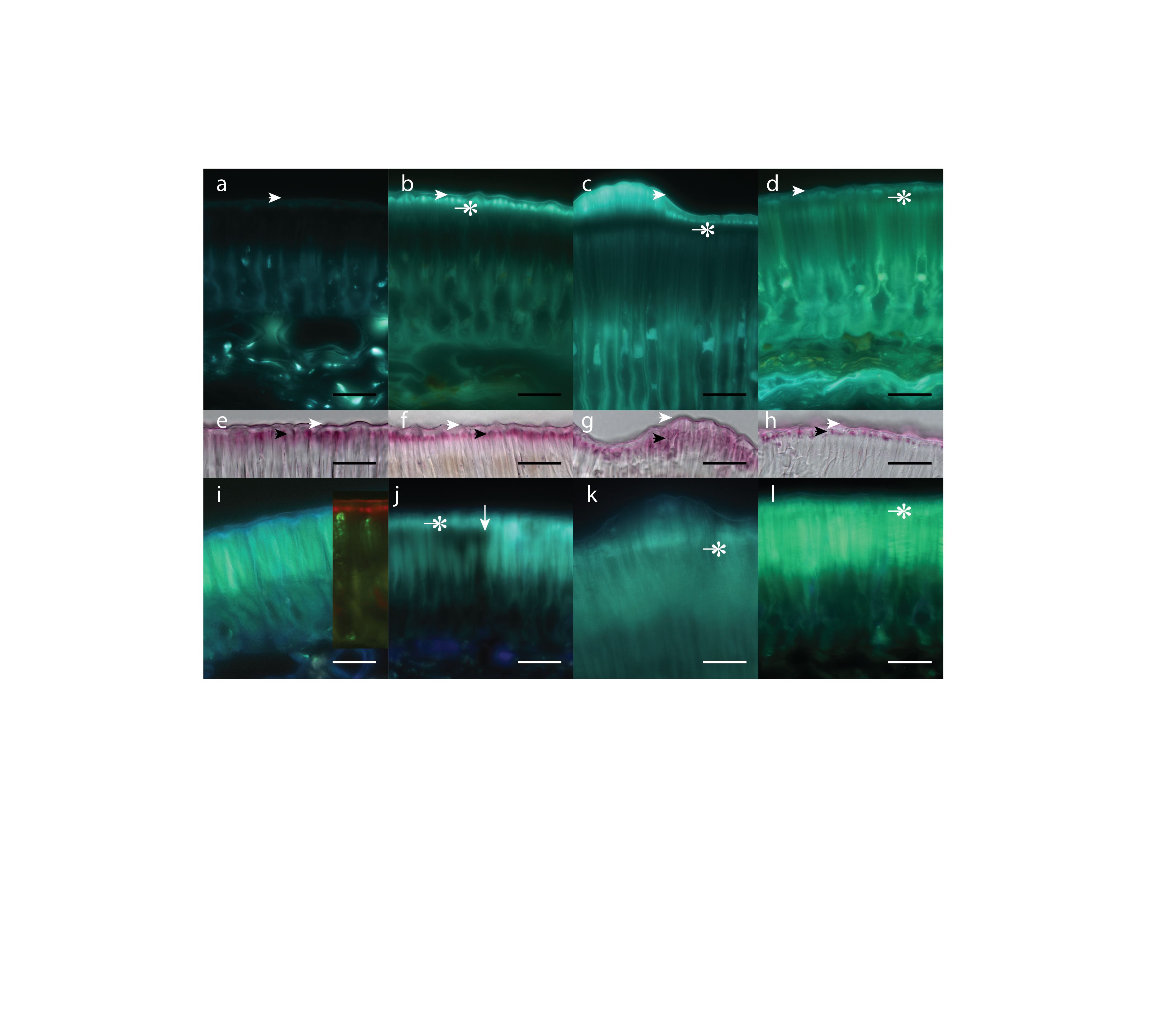

Supplement: Figure S2 — Seed coat transverse sections from extrahilar region: Cameor (a), JI92 (b), JI64 (c), and VIR320 (d). UV excited autofluorescence (a–d): white arrow, cuticle; asterisk, light line. Sudan Red 7B staining of terminal parts of macrosclereids (e–h): white arrow, cuticle; black arrow, lipidic material different from the cuticle). Aniline blue fluorochrome staining under UV excitation (i–l): asterisk, light line; white arrow, the edge between pigmented and non-pigmented interface of JI92, inlay in (i) callose immunodetection (blue excitation); scale bars = 25 μm) [file Image2.JPEG]

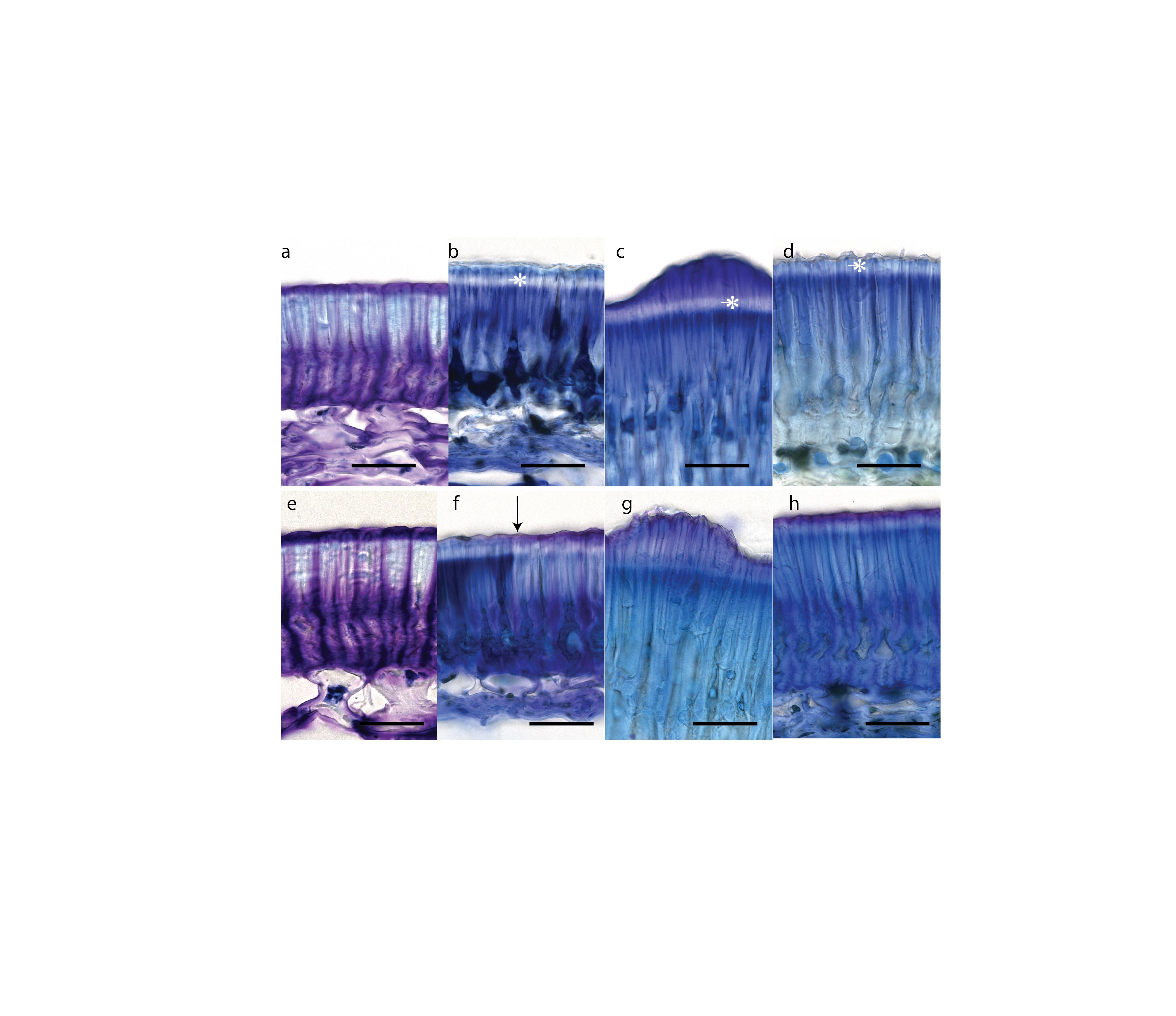

Supplement: Figure S3 — Seed coat transverse sections from extrahilar region: Cameor (a), JI92 (b), JI64 (c), and VIR320 (d). Metachromatic toluidine blue staining is indicative of high density of polyanionic surface (a–d): asterisk, light line. Toluidine blue staining after mild acid treatment (e–h): black arrow = the edge between pigmented and non-pigmented interface of JI92; scale bar = 50 μm. [file Image3.JPEG]

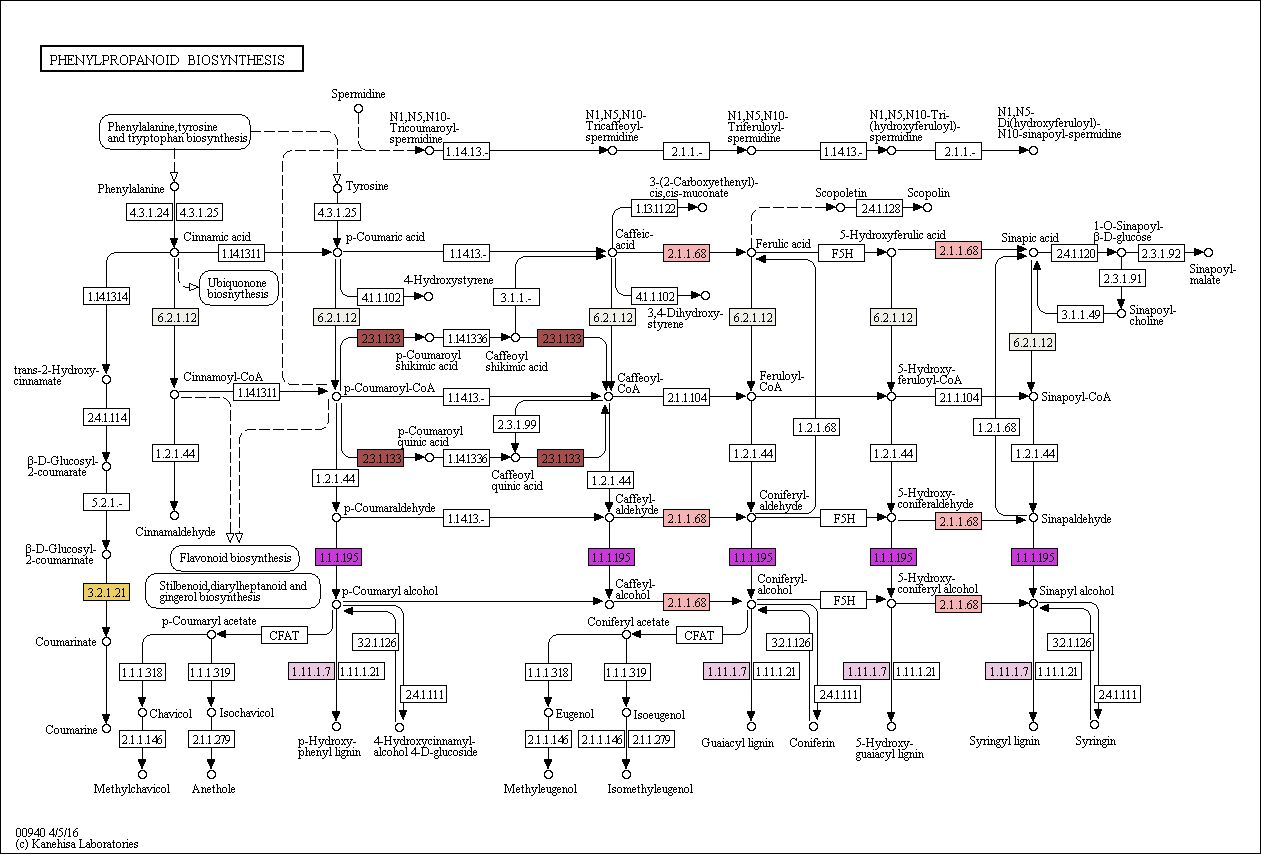

Supplement: Figure S4 — KEGG phenylpropanoid (A) and flavonoid (B) pathways of DEGs between dormant and nondormant seeds. [file Image4.JPEG]

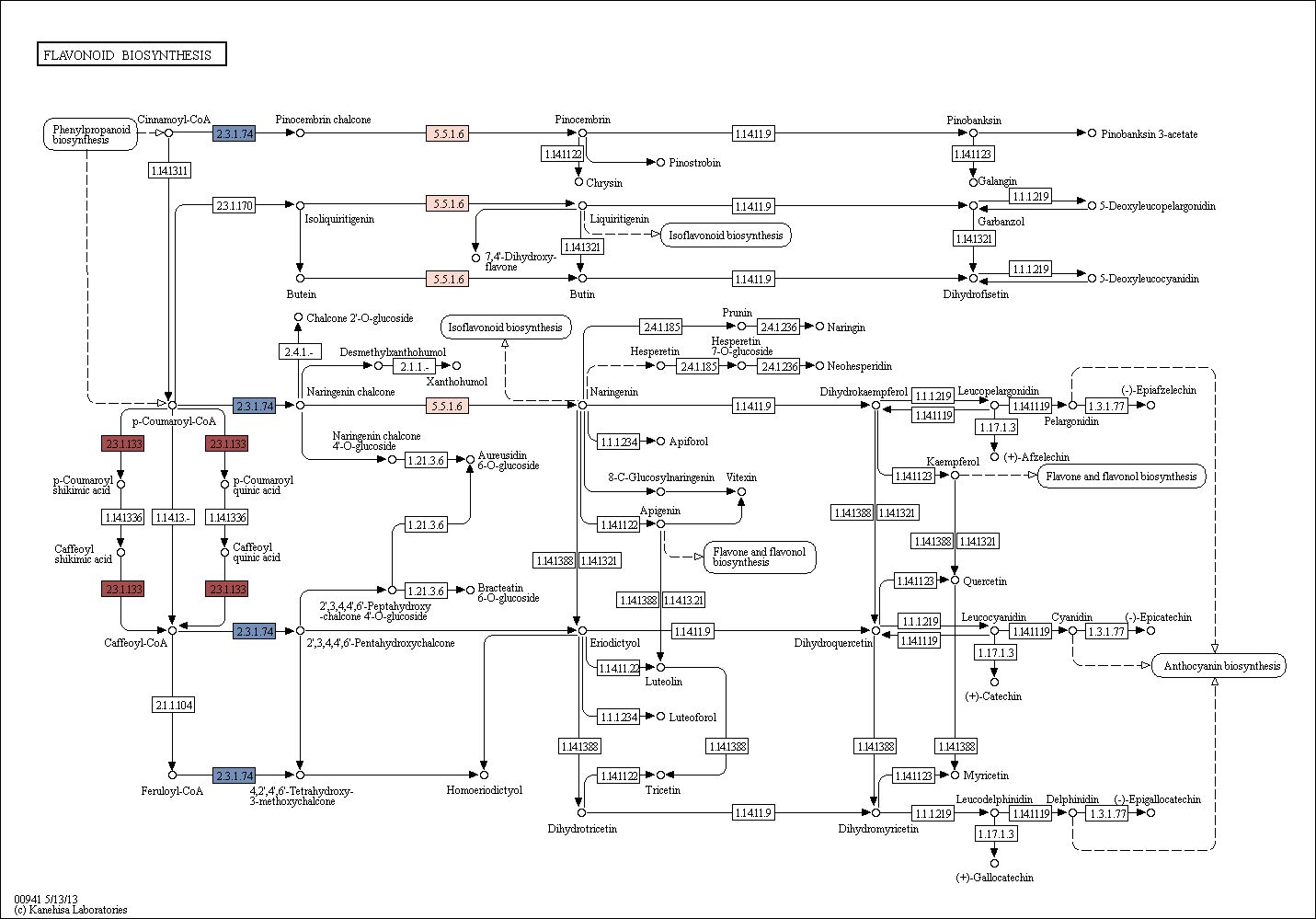

Supplement: Figure S5 — KEGG phenylpropanoid pathway of DEGs between dehiscent and indehiscent pods. [file Image5.JPEG]

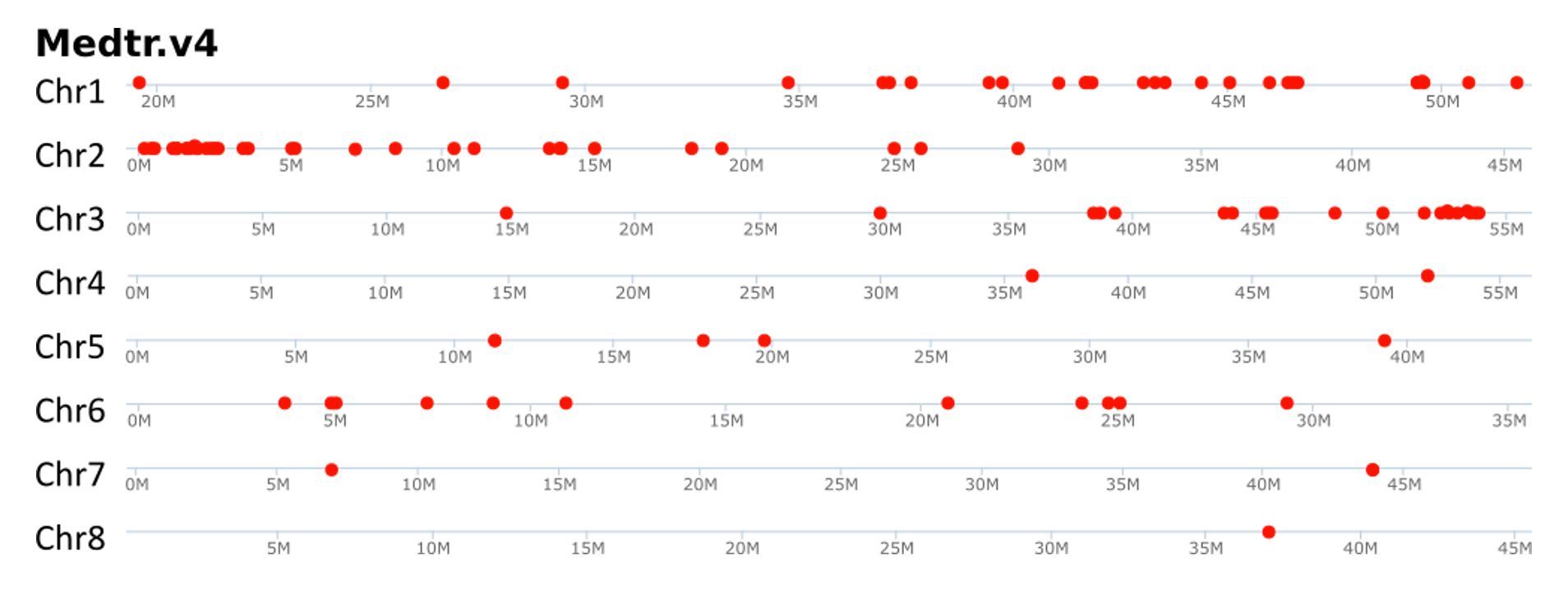

Supplement: Figure S6 — Strictly homozygous SNP between dehiscence and indehiscence RIL bulks mapped to the eight Medicago truncatula chromosomes. [file Image6.JPEG]
